# Prevalences of respiratory viruses and bacteria in Western Canadian commercial feedlot calves detected using a single metagenomic sequencing protocol vary during the first two weeks of arrival and by age group

**DOI:** 10.3389/fvets.2025.1704412

**Published:** 2026-02-05

**Authors:** Emmanuel Donbraye, Lianne McLeod, Claire N. Carson, Zhijian Chai, Stacey R. Lacoste, Emily K. Herman, E. Luke McCarthy, Janet E. Hill, Nathan E. N. Erickson, Colleen Pollock, Matthew G. Links, Simon J. G. Otto, Sheryl Gow, Paul Stothard, John R. Campbell, Cheryl L. Waldner

**Affiliations:** 1 Department of Large Animal Clinical Sciences, Western College of Veterinary Medicine, University of Saskatchewan, Saskatoon, SK, Canada; 2 Department of Laboratory Medicine, Royal University Hospital, Saskatoon, SK, Canada; 3 Department of Biochemistry and Medical Genetics, Max Rady College of Medicine, University of Manitoba, Winnipeg, MB, Canada; 4 Department of Animal and Poultry Science, College of Agriculture and Bioresources, University of Saskatchewan, Saskatoon, SK, Canada; 5 Department of Veterinary Microbiology, Western College of Veterinary Medicine, University of Saskatchewan, Saskatoon, SK, Canada; 6 HEAT-AMR (Human-Environment-Animal Transdisciplinary AMR) Research Group, School of Public Health, University of Alberta, Edmonton, AB, Canada; 7 Centre for Healthy Communities, School of Public Health, University of Alberta, Edmonton, AB, Canada; 8 Canadian Integrated Program for Antimicrobial Resistance Surveillance, Public Health Agency of Canada, Saskatoon, SK, Canada; 9 Department of Agricultural, Food, and Nutritional Science, Faculty of Agricultural, Life, and Environmental Sciences, University of Alberta, Edmonton, AB, Canada

**Keywords:** bovine respiratory viruses, bovine respiratory bacteria, antimicrobial resistance genes, bovine respiratory disease, long-read metagenomic sequencing, feedlot calves

## Abstract

**Introduction:**

Detection of pathogens associated with bovine respiratory disease (BRD) typically involves several laboratory tools, with results limited to a defined list of targets. This study adapted a previously reported method for metagenomic sequencing of nasal swabs to describe sequencing data from BRD associated viruses. Changes in virus composition were identified between arrival to a feedlot and 14 days on feed (DOF). These data were also assessed for the simultaneous characterization of bacteria and antimicrobial resistance genes (ARGs).

**Methods:**

Nasal swabs were obtained from fall-placed calves (FPC) and yearlings (YRL) from western Canadian commercial feedlots. Evidence of respiratory viruses were identified by sampling 380 animals during processing on arrival to the feedlot and again after 14 DOF using Nanopore metagenomic sequencing.

**Results:**

Twenty-one distinct viruses from 12 viral families were identified, with multiple viruses detected in most samples. In FPC arrival samples, the most common BRD associated viruses were bovine rhinitis B virus (BRBV; 46%), bovine coronavirus (BCoV; 32%), influenza D virus (IDV; 17%), bovine respiratory syncytial virus (BRSV; 8.5%), and bovine parainfluenza virus 3 (BPIV-3; 4.2%). The prevalences of bovine herpesvirus type 1 (BoHV-1; 2.7%), BPIV-3 (12%), BRSV (26%), and IDV (51%) were higher in 14 DOF samples compared to arrival samples (*p* < 0.05). Bovine viral diarrhea virus 1 (BVDV-1) and 2 (BVDV-2) were rarely detected at either time. The most prevalent viruses detected in YRL arrival samples were BRBV (42%), BRSV (39%), BPIV-3 (20%), IDV (16%), BCoV (12%), and BVDV-2 (7.5%). The prevalences of BRSV (60%), BPIV-3 (39%), and BVDV-2 (17%) were higher in 14 DOF samples than arrival samples (*p* < 0.05). BRSV (OR 7.0, 1.7–29) and BPIV-3 (OR 5.7, 1.5–21) were more likely to be detected in arrival samples from YRL than FPC (*p* = 0.01). In 14 DOF samples, BPIV-3 (OR 4.9, 1.3–19, *p* = 0.02) and BVDV-2 (OR 13, 2.0–83, *p* = 0.01) were identified more frequently in YRL than FPC. These data allowed the identification of respiratory bacteria and 33 ARGs in parallel with assessment of the viral components. The most prevalent bacteria detected in FPC at arrival were *Mannheimia haemolytica* (35%), *Histophilus somni* (35%) and *Pasteurella multocida* (23%). Detection of *M. haemolytica* increased at 14 DOF (*p* = 0.02), while *P. multocida* detection decreased (*p* = 0.03). At both arrival and 14 DOF in YRL, *M. haemolytica* was the most prevalent bacterium, followed by *P. multocida* and *H. somni* with no significant differences between arrival and 14 DOF samples. ARGs were detected more frequently in the 14 DOF samples than at arrival for both FPC (*p* = 0.03) and YRL (*p* = 0.01). The most commonly detected ARGs were associated with resistance to lincosamides and aminoglycosides; however, ARGs associated with other antimicrobials used in cattle including tetracyclines were also identified.

**Discussion:**

Changes in the prevalence of BRD associated viruses early in the feeding period reflect transmission and the potential risk of developing the disease. Frequent detection of BCoV, BRSV, and BPIV-3 in newly arrived feedlot cattle suggests the need for improved vaccination before shipping or limitations in existing commercial vaccine preparations.

## 1 Introduction

Bovine respiratory disease (BRD) has historically accounted for most of the morbidity and mortality in North American feedlot cattle (1, 2). BRD is the primary reason for injectable antimicrobial use (AMU) in feedlots and also one of the foremost reasons for AMU in cow-calf herds (3, 4).

The causes of BRD are complex and involve a combination of environmental factors, calf management, stress, as well as bacterial and viral agents that all play a role in the onset of disease (5). Viral infection can result in clinical BRD (6); however, many viruses can make a calf susceptible to secondary bacterial infection. Most bacteria associated with BRD are considered commensal organisms of the upper respiratory tract in healthy calves, but can become opportunistic pathogens when the host immune system is compromised (7). During primary infection, viral pathogens can damage the respiratory tract epithelium or cause immunosuppression, thereby rendering the animal more susceptible to bacterial infection (5). Bacteria commonly associated with BRD include *Mannheimia haemolytica*, *Pasteurella multocida*, *Histophilus somni*, *Mycoplasma bovis*, and occasionally *Bibersteinia trehalosi* (8) [note the genus name of *Mycoplasma* has recently changed to *Mycoplasmopsis*; (9)]. Viral agents historically associated with BRD and typically contained in commercial respiratory vaccines include bovine respiratory syncytial virus (BRSV; *Bovine orthopneumovirus*) (10), bovine herpesvirus type 1 (BoHV-1; *Bovine alphaherpesvirus* 1) (11), bovine viral diarrhea virus (BVDV-1, *Pestivirus bovis* and BVDV-2, *Pestivirus tauri*) (12), and bovine parainfluenza virus type 3 (BPIV-3; Bovine respirovirus-3) (13).

Newly weaned calves are at greatest risk of BRD during the first few weeks following feedlot arrival. This risk is heightened because in a feedlot calves are typically comingled with cattle from other sources and further stressed from days in the marketing system with inconsistent access to feed and water (14–18). These calves are prone to viral infections and subsequently shed viruses, infecting pen mates, and increase the risk of infection with opportunistic bacteria and therefore the need for antimicrobial treatment (19, 20).

Most Canadian feedlot calves are sourced from cow-calf herds in western Canada, many of which could have been vaccinated against BVDV-1, BVDV-2, BoHV-1, BRSV, and BPlV-3 (21, 22). While the use of BRD vaccines in nursing calves appears to be increasing (21), vaccination practices in cow-calf herds have historically been inconsistent and poorly documented. Calves successfully vaccinated, including both prime and boost doses, against these viruses before weaning should be less likely to shed virus when they arrive at the feedlot (23); however, data on the frequency of virus shedding by calves at feedlot arrival in western Canada are limited (24).

Despite increasing vaccine use for BVDV-1, BVDV-2, BoHV-1, BRSV, and BPlV-3 in cow-calf herds, BRD remains an important health challenge for feedlots (25). As the vaccination status of arriving calves is typically unknown, feedlots vaccinate at arrival processing despite evidence of limited efficacy due to prior viral exposure during sale and transport (26). As viruses beyond those in commercial vaccines are implicated in BRD, further vaccine development is needed (20, 27). While vaccines are available for bovine coronavirus-associated (BCoV) calf diarrhea, no commercial respiratory vaccines for BCoV are available in Canada or the USA (28). Other recognized viruses, such as influenza D virus (IDV) (29) and, more recently, influenza A virus, might also warrant new vaccine development (30).

The advent of metagenomic sequencing has the potential to substantially transform viral diagnostics, enabling the direct detection of both known and unreported viruses in clinical samples (24, 31, 32). Consequently, metagenomics is being increasingly reported for virus surveillance, pathogen discovery, and viral disease outbreak detection (33–37). Metagenomic sequencing has facilitated the identification of novel or infrequently evaluated viruses in both healthy and sick animals, as well as humans (31, 35). Metagenomics has been particularly valuable for detecting viruses in animals with BRD without the need for targeted assays (32, 38–40). Among the various metagenomic sequencing technologies currently available, Oxford Nanopore Technologies (ONT) is noteworthy for long-read capabilities and cost effectiveness (41).

Recent progress in metagenomic sequencing technology has led to the development of protocols that can detect multiple known viruses associated with BRD as well as uncover a broader range of viruses potentially linked to the disease, such as bovine rhinitis B virus (BRBV) (32, 39), bovine adenovirus type 3 (BAdV-3) (39), and ungulate copiparvovirus type 1 (UCPV1) (42). Understanding the range of viruses present in cattle entering the feedlot as well as in the early feeding period could lead to the development of more effective strategies for the prevention and control of BRD in western Canada (32, 38).

Metagenomic investigations into the virome of the bovine respiratory tract have predominantly been conducted when calves exhibit clinical signs of BRD or upon their arrival at the feedlot (24, 32, 38–40, 43). No studies have yet examined changes in the early stages of the feeding period as compared to viruses present at arrival. Furthermore, existing viral metagenomic studies have overlooked BRD bacterial pathogens of interest and antimicrobial resistance genes (ARGs). Previous metagenomic studies with ONT have successfully identified both bacteria and ARGs of interest in similar samples but with protocols not optimized for detection of RNA viruses (44, 45). Understanding the diversity, prevalence, and dynamics of viruses and bacteria associated with BRD, including ARGs, with a single protocol could facilitate the development of more effective BRD prevention and control. Considering that commercial feedlots can accommodate hundreds or even thousands of cattle at a time, the need to enhance laboratory and bioinformatics capabilities to manage large sample volumes for surveillance and ultimately to inform decision-making is urgent.

The primary objective of this study was to describe the prevalence of viruses reported to be important to the development of BRD as well as other viruses previously detected in respiratory samples collected from feedlot cattle at arrival processing and again approximately 2 weeks later. The second objective was to describe changes in the prevalence of viruses associated with BRD in feedlot cattle early in the feeding period. The third objective was to explore the potential for exploiting sequencing data generated from this viral metagenomic analysis for the simultaneous detection of bacterial BRD pathogens and associated ARGs.

## 2 Materials and methods

### 2.1 Ethics statement

This study was conducted in accordance with the recommendations of the Canadian Council of Animal Care (CCAC) (46). An ethics protocol and standard operating procedure for nasal swab collection was developed and approved by the Animal Care and Use Committee at the University of Alberta (ACUC Livestock – University of Alberta AUP00004110) and shared with the Research Ethics Board at the University of Saskatchewan (USask AREB File Number 20220072) and the Animal Care Committee of Feedlot Health Management Services as a study partner.

### 2.2 Study population and sampling procedure

The study was carried out in collaboration with the Canadian Feedlot Antimicrobial Use and Antimicrobial Resistance Surveillance Program (CFAASP) (47) of the Canadian Integrated Program for Antimicrobial Resistance Surveillance (CIPARS) as part of a nationwide, longitudinal survey of antimicrobial resistance in feedlot cattle in Canada (48). The CIPARS program partnered with consulting veterinary practices that identified volunteers intended to represent the distribution of feedlot sizes and geographic locations within the Canadian feedlot industry. Only cattle arriving before December 31, 2022 as part of the annual CIPARS sample collection for culture and susceptibility testing were included. An additional nasal swab from each animal was collected for viral metagenomics.

Private veterinary clinics participating in the CIPARS program sampled cattle from 13 pens of fall-placed calves (FPC) and 6 pens of yearlings (YRL) arriving at 19 commercial feedlots (with one pen from each feedlot) in Alberta between September and December 2022 that agreed to the extra sample collection for this project. Typically, FPC would be recently weaned spring-born beef calves and YRL would be calves born the previous season that had been backgrounded and/or grazed as stockers prior to feedlot entry. Feedlots were anonymized and therefore no information was available regarding feedlot management, pen size, treatment histories, or arrival processing protocols including either vaccination or metaphylaxis. Sampling was conducted within CFAASP when FPC are at the highest risk of developing BRD.

Registered veterinary technicians with training in feedlot settings utilized conventional techniques to restrain FPC and YRL for the collection of nasal swabs. A single short nasal swab was collected for this virus study from a convenience sampling of 20 cattle from each pen at arrival of the cattle to the feedlots but prior to metaphylaxis. Nasal swabs were again collected from another convenience sample of 20 animals from the same pen after 14 days on feed (DOF), but not necessarily from the same individual cattle.

Following sampling, 3 cm of each swab tip was cut and placed in a tube containing 1 mL of liquid Amies transport media (CoPan Diagnostics, Carlsbad, CA, USA) and shipped on ice packs in coolers to the University of Saskatchewan. The samples were received cold, but not frozen, and were then stored at −80 °C, until processing.

### 2.3 Sample preparation

Samples were thawed at room-temperature in a water bath and then maintained on-ice during processing. Batches of 40 samples were processed with an extraction negative control (molecular biology grade water). Samples were centrifuged at 13,000 × g for 5 min to pellet bacteria, host cells, and debris. To degrade extracellular nucleic acids, and aid in host depletion, 500 μL of sample supernatant were combined with 34 μL of nuclease digestion master mix (6 units of TURBO DNase, 20 μL TURBO DNase buffer, 20 units RNase I; Invitrogen, Waltham, MA, USA) and incubated at 37 °C for 90 min. This was followed by the purification of viral nucleic acids using the QIAamp MinElute Virus Spin Kit (QIAGEN, Hilden, Germany), following the manufacturers’ instructions. Briefly, carrier RNA (catalogue number – 57714; QIAGEN, Hilden, Germany) was added and then proteins were digested with protease at 56 °C for 15 min, and then treated with 95% ethanol to halt enzyme activity. The lysates were held at room temperature for 5 min before being transferred to MinElute columns (QIAGEN, Hilden, Germany) prepared on vacuum manifolds. The bound sample was washed with 95% ethanol within the column. Following washing, samples were spun at 20,000 x g for 3 min and heated at 56 °C for 3 min to dry the membrane. Samples were eluted in 40 μL of AVE buffer by incubating for 5 min at room temperature and then spinning at 17,000 × g for 1 min.

SuperScript IV First-Strand Synthesis Kit (Applied Biosystems, Waltham, MA, USA) was used for reverse transcription. Ten μL of nucleic acids per sample were annealed with 3.5 μL of a random primer mastermix (1 μM FR26RV-N primer, 0.5 mM dNTPs, 0.42 μL of DEPC-treated water) at incubated at 65 °C for 5 min (49). Each sample was placed on ice for 1 min to stabilize the nucleic acid-primer complex, and then combined with 7 μL of reverse transcription master mix (4 μL Superscript IV 5 × buffer, 1 μL DTT, 1 μL RNase inhibitor, and 1 μL RTase; Invitrogen, Waltham, MA, USA) and incubated for 10 min each at 23, 55, and then 80 °C. After incubation, samples were placed on ice for 1 min and RNA was degraded by adding 1 μL of *E. coli* RNase H (5 U/μL) and incubation for 20 min at 37 °C.

Second-strand synthesis was performed using Sequenase DNA Polymerase (Applied Biosystems, Waltham, MA, USA) according to manufacturers’ instructions. cDNA synthesis was performed by adding 10 μL of Sequenase mastermix (2 μL 5 × Sequenase Buffer, 0.3 μL Sequenase enzyme, 7.7 μL RNase-free water; Applied Biosystems, Waltham, MA, USA) at room temperature and then incubated in a ramped fashion: slow ramp from 10 to 37 °C over 8 min, 37 °C for 8 min, 94 °C for 2 min, and finally 10 °C for 5 min. The reaction was then topped up with an additional 0.3 μL of Sequenase enzyme and 0.9 μL of dilution buffer and incubated under the same reaction conditions.

cDNA from each sample and extraction negative control was cleaned-up and size-selected with a ratio of 1:1 AMPure XP bead suspension by volume (Beckman Coulter, Brea, CA, USA) according to the manufacturer’s instructions, and eluted in a volume of 10 μL. Size selected cDNA (8 μL) was combined with 42 μL of amplification mastermix (1 × Standard Buffer, 100 μM dNTP, 1.5 mM MgCl2, 0.5 μM FR20RV primer {Allander, 2005 #1197}, 0.25 μL polymerase, NEB, Ipswich, MA, USA) and cycled with the following reaction conditions: denaturation for 10 min at 94 °C, followed by 40 cycles of 94 °C for 1 min, 65 °C for 1 min, and 72 °C for 3 min, followed by a final extension of 5 min at 72 °C.

### 2.4 Nanopore library preparation and sequencing

The amplified cDNA from each sample was purified and size selected with 0.4 × AMPure XP beads (Beckman Coulter, Brea, CA, USA) and normalized to 50 ng total DNA prior to library preparation. Library preparation was completed according to the Oxford Nanopore Technologies protocol “Ligation sequencing gDNA - Native Barcoding kit 96 V14” (SQK-NBD114.96, Oxford Nanopore Technologies, Oxford, UK) in a 96-well plate high-throughput library format with minor modifications to minimize the potential for any misclassification across barcoded samples or background barcode crosstalk (50, 51).

Barcode ligation was followed by the addition of 1 μL of EDTA (Invitrogen, Waltham, MA, USA), a 10 min room temperature incubation, and a 10 min 65 °C incubation. DNA samples, barcoded, were pooled together in groups of 20 from the same feedlot pen with three additional water (negative) controls per library preparation to detect barcode crosstalk that might occur during the library preparation and subsequent sequencing (52). Sample pools were sequenced as a pool on a flow cell (FLO-PRO114M). Negative controls for both the extraction and library preparation steps were processed together on a separate flow cell (FLO-PRO114M).

All flow cells (Oxford Nanopore Technologies, Oxford, UK) were loaded according to manufacturer recommendations. Sequencing was performed under contract by the Omics and Precision Agriculture Laboratory (OPAL; Saskatoon, SK, Canada) using a PromethION 24. Sequencing was performed for 48 h with default run parameters and base-called using the high-accuracy model with a Q-score cutoff of 9.

### 2.5 Bioinformatics analysis

Github access to the bioinformatics details is provided in the data availability statement. Data from the fastq_pass folder from MinKNOW were processed with Porechop v0.2.4 (53) to remove primers or adapters (using default parameters). NanoFilt (version 2.8.0) was used to discard reads shorter than 200 bp and NanoStat (version 1.6.0) provided statistics about the distribution of read length by total base pairs per sample (54).

The taxonomic classification of reads was achieved using Kraken 2 (version 2.1.2) with a confidence score threshold of 0.05 (“--confidence 0.05”) (55). A custom database was used for Kraken 2 classification, which included bacterial, viral, and archaeal subsets of the November 2023 RefSeq database (56) as well as the *Bos taurus* ARS-UCD1.2_Btau5.0.1Y genome assembly available at https://sites.ualberta.ca/~stothard/1000_bull_genomes/ (44, 57). Typically, sequences classified as host would be removed before downstream processing; however, a small population of chimeric *B. taurus* bacterial reads (< 0.1% of all reads) was detected. A custom program, kmer_filter.py, was written to retrieve host-classified reads that met a threshold of 25% non-host sequence using Kraken 2 k-mer identity and included these reads as potentially non-host data for downstream processing. The rationale behind this step was to cast the widest possible net for ARG detection, even if a small amount of host sequence remained. Host-filtered reads (i.e., those not similar to *B. taurus* taxid 9,913) were extracted using the KrakenTools v1.2 (58) utility extract_kraken_reads.py, and these were added to the chimeric reads using a combination of bash utilities and the BBTools v38.86 (59) filterbyname.sh script. Bracken v2.7 (60) with a minimum read length of 200 bp (“--read-length 200”) was used to improve the species-level estimation of abundance reported by Kraken 2. Reads classified as host were removed from further consideration. A custom script, report_taxon_read_lengths.py, added additional context to the Bracken results, including the total amount of sequence in base pairs reported for each species (including child taxa) and the fraction of total classified sequence.

To identify reads containing ARGs, non-host reads were first converted from FASTQ to FASTA format using Seqtk v1.3 (61). AMRFinderPlus v3.11.18 (62) and Abricate v1.0.0 (63) were used to search for genes involved in virulence, biocide, heat, metal, and acid resistance. AMRFinderPlus was run against the NCBI Bacterial Antimicrobial Resistance Reference Gene Database (version 2023-11-15.1) with “--plus --coverage_min 0.8 --ident_min 0.8” to identify the plus genes while requiring a minimum coverage and minimum identity of 80%. Abricate used two databases: the NCBI Bacterial Antimicrobial Resistance Reference Gene Database (version 2023-11-15.1) and the Comprehensive Antibiotic Resistance Database (CARD) (64, 65) version 3.2.8 using default parameters (80% minimum percent identity and percent coverage). Results generated by ARG searching with the NCBI and CARD databases were merged based on gene name and start/stop coordinates. Once merged, the CARD gene names were preferentially used in downstream reports.

For all viral data and associated statistical analyses, the read counts were adjusted based on the corresponding read counts in the water controls. The average read count of the water controls for each flow cell (which corresponded to a pen) was subtracted from the observed read count for each sample to account for any potential misclassification across barcoded samples or barcode crosstalk (50, 51). For example, if the average read count for a specific virus for the water controls from a flow cell was 3, then the adjusted read count for that virus from each sample from that flow cell would be the observed sample read count minus 3.

Statistical analyses for the viral data were repeated using two alternative approaches: one without any adjustment for water controls and the other using the median counts of the water controls (rather than the average) for adjustment. A sensitivity analysis was conducted to evaluate the effects of the three methods for addressing potential contamination or barcode crosstalk. The three methods (with no adjustment, with adjustment for the mean, and with adjustment for the median of the water controls) were also repeated for the bacterial data.

### 2.6 Statistical analysis

The analysis pipeline of the present study was designed with the capacity to identify, directly characterize, and summarize detected reads for viruses and then bacteria of interest with an emphasis on those previously reported as associated with BRD. Initial descriptive statistics included the overall read count for each virus previously identified in respiratory samples from cattle and the proportion of samples that had at least one detected viral read for each virus.

All subsequent statistical analyses were limited to 10 viruses that met the following inclusion criteria: five BRD viruses with commercial respiratory vaccines (BRSV, BoHV-1, BVDV-1, BVDV-2, BPIV-3) in common use in North America (20) as well as five other viruses recently reported to exhibit a significant association with BRD (IDV, BCoV, BRBV, BAdV-3, UCPV1) (6, 32, 38, 39, 66). Only viruses with reported potential relationship to respiratory diseases in cattle were considered further.

The read count used as a cutoff to identify positive samples for common BRD-associated viruses (BoHV-1, BCoV, BPIV-3, IDV, and BRSV) was previously determined using Bayesian latent class modeling (BLCM) referencing qPCR results to optimize both sensitivity and specificity, with a minimum threshold of 0.90 for metagenomic specificity (67). Read counts were used to summarize the viral data rather than total base pairs given the relatively limited range of individual read lengths and the relatively small read counts for many of the viruses detected. The earlier BLCMs (67) used JAGS software (68) and the runjags package (69) in R (R Foundation for Statistical Computing, Vienna, Austria). Samples were classified as positive for BRD-associated viruses based on ≥ 1 read (BoHV-1, BRSV, and IDV) except for BPIV-3 (≥ 5 reads) and BCoV (≥ 30 reads). Read count cutoffs were developed for viruses where a qPCR assay was available to practitioners at the regional diagnostic laboratory at the time of the study. The qPCR was not type specific for BVDV-1 and BVDV-2. Viruses with no evidence-based read count cutoff (BVDV-1, BVDV-2, BRBV, BAdV-3, UCPV-1) were reported separately from viruses for which a cutoff was available. Prevalences of these viruses were based on the detection of ≥ 1 read per sample.

Generalized estimating equations (GEEs) were used to estimate differences in the detection of each of the viruses previously associated with BRD: (1) between samples collected at arrival processing and 14 DOF for FPC and YRL and (2) between FPC and YRL at each sample time point. The GEE models accounted for clustering of observations within feedlot pens with a repeated term for pen assuming robust variance, an exchangeable correlation structure, a binomial distribution, and logit link function (StataSE version 16.1, College Station, TX, USA). Viruses were coded as present or absent based on the count of reads detected in the sample as described above. Time of sampling (arrival processing versus 14 DOF) and the age of cattle (FPC versus YRL) were considered as fixed effects. Results were reported as odds ratios (OR) with 95% confidence intervals (95% CI). Statistically significant results were defined as those with *p <* 0.05.

Descriptive statistics were also completed for BRD-associated bacteria and ARGs. GEEs as described above were used to assess differences in the detection of each bacterial species previously linked to BRD: (1) between samples collected at arrival processing and 14 DOF for FPC and YRL and (2) between FPC and YRL at each sample time point. Samples were classified as positive for BRD-associated bacteria based on cutoff values for species-specific read counts adjusted for the mean of the water controls and informed by BLCM as previously described (67).

## 3 Results

### 3.1 Sample preparation

Overall, 760 samples were collected: 20 nasal swabs per pen from 13 pens of FPC and six pens of YRL across 19 commercial feedlots in western Canada, at arrival processing (sample time 1) and again from the same pens at a median of 14 DOF (sample time 2). Timing of the second sample collection ranged from 10 to 23 DOF. The mean pen arrival weight was 266 kg (43 kg standard deviation (SD)) for the 13 pens of FPC and 399 kg (66 kg SD) for the six pens of YRL. The distribution of sizes for the 19 feedlots from which the pens were sampled was as follows: 1000–5,000 cattle = 1, 5,001–10,000 cattle = 5, 10,001–20,000 cattle = 5, 20,001–30,000 cattle = 6, and ≥ 30,000 cattle = 2.

### 3.2 Viral metagenomics

After demultiplexing, trimming, and quality filtering, 841.6 million reads were generated with a median quality score per sample ranging from 13.1 to 17.6 (median 14.1) and a median read length per sample ranging from 232 to 790 bp (median 420 bp). Following the removal of host-related sequences (691.7 million reads or 82.2% of the data), 149.8 million reads remained. Of the total reads, 1.95 million reads were classified as viral, 3.4 million reads as bacterial, 12,166 reads as archaeal, and 144.5 million reads as unclassified or classified ambiguously.

After adjusting read counts for the means of the water controls, 1.82 million reads were associated with the respiratory viruses of interest (Table 1). Twenty-one unique viruses from 12 viral families were identified that had previously been recognized in respiratory samples from cattle (Table 1; Supplementary Table S1). The total number of reads classified for each virus ranged from seven (BAdV-3) to > 1,000,000 (BCoV).

**Table 1:** Respiratory viruses detected in 760 nasal swabs collected from fall-placed calves and yearlings located in 19 western Canadian commercial feedlots/pens at the time of arrival processing and at 14 days on feed (DOF): summary of raw data from all samples.

Virus name	Family	Total reads from all samples	Proportion of samples with ≥ 1 read	Median (max) sample median read lengths (bp)	Previous reports of virus in cattle	Associated with BRD
BCoV	*Coronaviridae*	1,164,174	52.0%	875 (2313)	Zhang et al. (32), Ellis (96)	Yes
BRBV	*Picornaviridae*	59,307	39.5%	683 (1571)	Ng et al. (39)	Yes
IDV	*Orthomyxoviridae*	442,020	31.1%	773 (2709)	Hause et al. (27)	Yes
BPIV-3	*Paramyxoviridae*	63,315	33.7%	846 (2709)	Mitra et al. (38)	Yes
BRSV	*Pneumoviridae*	3,285	27.4%	595 (1690)	Mitra et al. (38)	Yes
UBPV6	*Parvoviridae*	51,477	24.7%	764 (2164)	Ambrose et al. (40)	No
UCPV1	*Parvoviridae*	506	10.5%	715 (1651)	Jager et al. (42)	Yes
UCPV5	*Parvoviridae*	1,639	9.2%	748 (1519)	Ambrose et al. (40)	No
UEPV1	*Parvoviridae*	29,284	8.7%	747 (2195)	Weber et al. (100)	No
BEV	*Picornaviridae*	1,177	7.0%	707 (1704)	Mitra et al. (38)	No
UTPV1	*Parvoviridae*	900	7.0%	792 (2192)	Zhang et al. (43)	No
BVDV-2	*Flaviviridae*	73	4.7%	867 (2577)	Zhang et al. (43)	Yes
BNV1	*Tobaniviridae*	286	4.3%	727 (1447)	Ambrose et al. (40)	No
BoTV	*Tobaniviridae*	74	4.1%	934 (2533)	Ambrose et al. (40)	No
BoPV	*Papillomaviridae*	102	2.8%	655 (2172)	Ambrose et al. (40)	No
BoPyV2	*Polyomaviridae*	35	2.6%	682 (2355)	Hierweger et al. (104)	No
UBPV1	*Parvoviridae*	37	2.4%	604 (1901)	Zhang et al. (43)	No
BoHV-1	*Herpesviridae*	22	0.9%	412 (819)	Ambrose et al. (40)	Yes
ICV	*Orthomyxoviridae*	21	0.8%	682 (847)	Zhang et al. (32)	No
BVDV-1	*Flaviviridae*	8	0.8%	568 (848)	Zhang et al. (32)	Yes
BAdV-3	*Adenoviridae*	7	0.8%	635 (1134)	Zhang et al. (32)	Yes

See Supplementary Table S1 for general virus names, taxonomic names, taxonmic ID and genome size.

### 3.3 Prevalences of BRD-associated viruses in nasal samples from FPC

Summary data in Table 2 indicate BRBV was the most prevalent virus in FPC at arrival processing, detected in 46% of nasal samples. BCoV was the second-most prevalent virus detected (32%) and most prevalent virus with a BLCM-based read count cutoff, followed by IDV (17%) and BRSV (8.5%). The prevalences of BPIV-3 (4.2%) and BVDV-2 (1.2%) were lower. Metagenomic sequencing did not detect either BVDV-1 or BoHV1 in any of the FPC arrival samples.

**Table 2:** Differences in prevalence of ten viruses associated with bovine respiratory disease detected in 520 nasal swabs collected from fall-placed calves (FPC) in western Canadian commercial feedlots at arrival processing and at 14 days on feed (DOF).

Samples from fall-placed calves											
Virus	Sample time 1: arrival processing (*n* = 260)				Sample time 2: 14 DOF (*n* = 260)				14 DOF samples compared to arrival samples		
	No. pos. samples	Prevalence	95% CI	No. reads	No. pos. samples	Prevalence	95% CI	No. reads	Odds ratio	95% CI	*p*-value
Viruses with cutoff for positive status based on BLCM (67)											
BCoV	82	32%	11–52%	1,152,758	27	10%	4.2–17%	4,364	0.25	0.09–0.68	**0.01**
IDV	44	17%	5.9–28%	17,309	133	51%	36–67%	415,856	5.14	2.33–11.3	**<0.001**
BRSV	22	8.5%	0.6–16%	78	67	26%	9.4–42%	2,411	3.76	1.32–10.7	**0.01**
BPIV-3	11	4.2%	0.5–8.0%	2,114	30	12%	2.9–20%	59,393	2.95	1.46–5.96	**<0.001**
BoHV-1	0	0%	0–1.4%	0	7	2.7%	0–5.9%	22	9.81	1.46-∞	**0.02**
Viruses for which there are no BLCM-informed cutoffs – Sample positive if ≥ 1 read											
BRBV	119	46%	34–58%	50,136	99	38%	26–50%	4,388	0.73	0.48–1.10	0.13
UCPV-1	21	8.1%	4.9–11%	206	45	17%	10–24%	282	2.38	1.42–4.00	**0.001**
BVDV-2	3	1.2%	0–3.4%	4	4	1.5%	0–3.8%	5	1.34	0.65–2.74	0.43
BAdV-3	3	1.2%	0–2.3%	3	1	0.4%	0–1.1%	2	0.33	0.03–3.43	0.35
BVDV-1	0	0%	0–1.4%	0	0	0%	0–1.4%	0	Not estimable		0.99

The cutoffs used to determine virus-positive samples were based on ≥ 1 read for all viruses except BPIV-3 (≥ 5 reads) and BCoV (≥ 30 reads).

FPC: fall-placed calves; BCoV: bovine coronavirus; BAdV-3: bovine adenovirus 3; BoHV-1: bovine herpesvirus 1; BPIV-3: bovine parainfluenza virus 3; BRBV: bovine rhinitis B virus; BRSV: bovine respiratory syncytial virus; BVDV-1: bovine viral diarrhea virus 1; BVDV-2: bovine viral diarrhea virus 2; IDV: influenza D virus; UCPV1: ungulate copiparvovirus 1; No.: number; POS: positive; CI: confidence interval; BLCM: Bayesian latent class models. Comparable tables were produced based on reads not adjusted for water controls (Supplementary Table S2A) and reads adjusted for the medians of the water controls (Supplementary Table S3A). The bold values indicate associations that are statistically significant.

IDV was the most prevalent virus detected in FPC 14 DOF samples (51%), followed by BRBV (38%), BRSV (26%), and BPIV-3 (12%). BVDV-1 was also not detected in any of the FPC 14 DOF samples. The prevalence of BRSV was higher in 14 DOF samples compared to arrival samples (*p =* 0.01), as were the prevalences of IDV (*p <* 0.001), BPIV-3 (*p <* 0.001), BoHV-1 (*p =* 0.02), and UCPV-1 (*p <* 0.001). In contrast, the prevalence of BCoV was lower at 14 DOF than at arrival (*p =* 0.01). The prevalences of other important core respiratory vaccine viruses remained relatively unchanged 2 weeks following feedlot arrival (BVDV-1 – 0%, BVDV-2 – 1.5%).

### 3.4 Prevalences of BRD-associated viruses in YRL nasal samples

Summary data in Table 3 indicate the most common viruses detected in samples collected from YRL at arrival processing were BRBV (42%), BRSV (39%), BPIV-3 (20%), and IDV (16%). However, BRSV was the most prevalent virus detected in YRL 14 DOF samples (60%), followed by BPIV-3 (39%), IDV (33%), and BRBV (27%). The prevalences of BRSV (*p =* 0.001), BPIV-3 (*p <* 0.001), and BVDV-2 (*p <* 0.001) were higher in YRL 14 DOF samples compared to arrival samples.

**Table 3:** Differences in prevalence of ten viruses associated with bovine respiratory disease detected in 240 nasal swabs collected from yearlings (YRL) in western Canadian commercial feedlots at the time of arrival processing and at 14 days on feed (DOF).

Samples from yearlings											
Virus	Sample time 1: arrival processing (*n* = 120)				Sample time 2: 14 DOF (*n* = 120)				14 DOF samples compared to arrival samples		
	No. pos. samples	Prevalence	95% CI	No. reads	No. pos. samples	Prevalence	95% CI	No. reads	Odds ratio	95% CI	*p-*value
Viruses with cutoff for positive status based on BLCM (67)											
BRSV	47	39%	15–63%	174	72	60%	26–94%	622	2.33	1.44–3.78	**0.001**
BPIV-3	24	20%	4.8–35%	333	47	39%	13–65%	1,180	2.56	1.80–3.68	**<0.001**
IDV	19	16%	0–38%	126	40	33%	5.3–61%	8,729	2.66	0.47–14.9	0.27
BCoV	14	12%	0–23%	2,610	21	18%	0–43%	2,773	1.61	0.57–4.50	0.37
BoHV-1	0	0%	0–3.0%	0	0	0%	0–3.0%	0	Not estimable		0.99
Viruses for which there are no BLCM informed cutoffs – Sample positive if ≥ 1 read											
BRBV	50	42%	14–70%	4,338	32	27%	1.3–52%	445	0.51	0.13–2.01	0.34
BVDV-2	9	7.5%	0.9–14%	14	20	17%	1.3–32%	50	2.47	1.87–3.26	**<0.001**
UCPV1	5	4.2%	0.5–24%	6	9	7.5%	1.8–13%	12	1.86	0.42–8.32	0.41
BVDV-1	2	1.7%	0–3.7%	2	4	3.3%	0–7.5%	6	2.03	0.43–9.66	0.37
BAdV-3	0	0%	0–3.0%	0	2	1.7%	0–3.6%	2	2.43	0.19-∞	0.50

The cutoffs used to determine virus-positive samples were based on ≥ 1 read for all viruses except BPIV-3 (≥ 5 reads) and BCoV (≥ 30 reads).

YRL: yearlings; BCoV: bovine coronavirus; BAdV-3: bovine adenovirus 3; BoHV-1: bovine herpesvirus 1; BPIV-3: bovine parainfluenza virus 3; BRBV: bovine rhinitis B virus; BRSV: bovine respiratory syncytial virus; BVDV-1: bovine viral diarrhea virus 1; BVDV-2: bovine viral diarrhea virus 2; IDV: influenza D virus; UCPV1: ungulate copiparvovirus 1; No.: number; CI: confidence interval; BLCM: Bayesian latent class models. Comparable tables were produced based on reads not adjusted for water controls (Supplementary Table S2B) and reads adjusted for the medians of the water controls (Supplementary Table S3B). The bold values indicate associations that are statistically significant.

### 3.5 Detection of multiple viruses in FPC and YRL nasal samples

**Figure 1:**
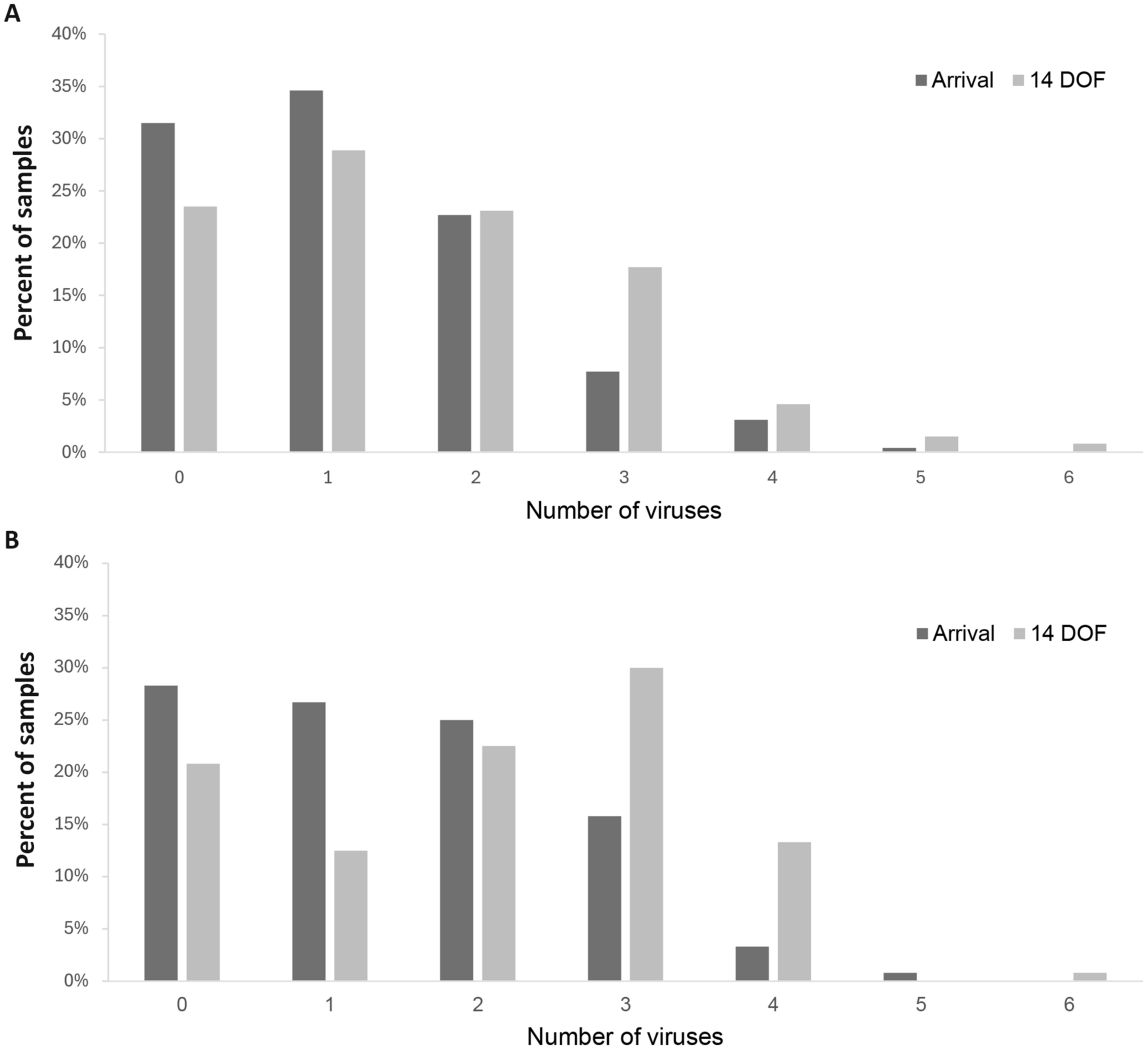
Frequency of codetection in individual samples for the ten identified BRD-associated viruses in fall-placed calves **(A)** from 13 feedlot pens at arrival processing (*n* = 260) and at 14 days on feed (DOF) (*n* = 260) (Table 2) and fall-placed yearlings **(B)** from 6 feedlot pens at arrival processing (*n* = 120) and at 14 days on feed (DOF) (*n* = 120) (Table 3) in western Canadian feedlots.

Figure 1A shows one or more of the 10 BRD-associated viruses were detected in most FPC samples collected at arrival processing (69%) and at 14 DOF (77%). Detection of a single virus occurred in 35% of FPC arrival samples and 29% of FPC 14 DOF samples. For FPC arrival samples in which more than one BRD virus was detected, typically two (23%) of the BRD-associated viruses were detected, while two (23%) or three (18%) viruses were more common in 14 DOF samples.

In YRL, one or more BRD viruses were detected in 72% of arrival samples and 79% of 14 DOF samples (Figure 1B). Detection of a single virus occurred in 27% of YRL arrival samples and 13% of YRL 14 DOF samples, followed by detection of two viruses in 25% of arrival samples and 23% of 14 DOF samples.

### 3.6 Differences in the prevalence of viruses in arrival samples between YRL and FPC

A comparison of datasets in Table 4 shows that, within the arrival samples, BRSV (*p =* 0.01) and BPIV-3 (*p =* 0.01) were more commonly detected in YRL than FPC. No other significant differences in the prevalence of individual respiratory viruses between YRL and FPC in the arrival samples were noted. BoHV-1 was not detected in either the YRL or FPC arrival samples.

**Table 4:** Differences in prevalence of ten viruses associated with bovine respiratory disease detected in samples collected at arrival processing between fall-placed calves (FPC) and yearlings (YRL) in western Canadian feedlots.

Virus	FPC arrival samples: (*n* = 260)		YRL arrival samples: (*n* = 120)		Arrival samples: YRL compared to FPC		
	Prevalence	95% CI	Prevalence	95% CI	Odds ratio	95% CI	*p-*value
Viruses with cutoff for positive status based on BLCM (67)							
BCoV	32%	11–52%	12%	0–23%	0.29	0.06–1.28	0.10
IDV	17%	5.9–28%	16%	0–38%	0.92	0.15–5.66	0.93
BRSV	8.5%	0.6–16%	39%	15–63%	6.97	1.66–29.3	**0.01**
BPIV-3	4.2%	0.5–8.0%	20%	4.8–35%	5.66	1.50–21.4	**0.01**
BoHV-1	0%	0–1.4%	0%	0–3.0%	Not estimable		0.99
Viruses for which there are no BLCM-informed cutoffs – Sample positive if ≥ 1 read							
BRBV	46%	34–58%	42%	14–70%	0.85	0.24–2.94	0.79
UCPV1	8.1%	4.9–11%	4.2%	0.5–24%	0.49	0.18–1.37	0.18
BVDV-2	1.2%	0–3.4%	7.5%	0.9–14%	6.95	0.79–61.2	0.08
BAdV3	1.2%	0–2.3%	0%	0–3.0%	0.56	0–5.25	0.64
BVDV-1	0%	0–1.4%	1.7%	0–3.7%	5.27	0.41-∞	0.20

The cutoffs used to determine virus-positive samples were based on ≥ 1 read for all viruses except BPIV-3 (≥ 5 reads) and BCoV (≥ 30 reads).

FPC: fall-placed calves; YRL: yearlings; BCoV: bovine coronavirus; BAdV-3: bovine adenovirus 3; BoHV-1: bovine herpesvirus 1; BPIV-3: bovine parainfluenza virus 3; BRBV: bovine rhinitis B virus; BRSV: bovine respiratory syncytial virus; BVDV-1: bovine viral diarrhea virus 1; BVDV-2: bovine viral diarrhea virus 2; IDV: influenza D virus; UCPV1: ungulate copiparvovirus 1; CI: confidence interval; BLCM: Bayesian latent class models. Comparable tables were produced based on reads not adjusted for water controls (Supplementary Table S2C) and reads adjusted for the medians of the water controls (Supplementary Table S3C). The bold values indicate associations that are statistically significant.

### 3.7 Differences in prevalence of viruses in 14 DOF samples between YRL and FPC

A comparison of datasets in Table 5 shows that, within 14 DOF samples, the prevalences of both BPIV-3 (*p =* 0.02), BVDV-1 (*p* = 0.02) and BVDV-2 (*p =* 0.01) were higher in YRL than FPC. No other significant differences in the prevalences of individual respiratory viruses between YRL and FPC in the 14 DOF samples were noted.

**Table 5:** Differences in prevalence of ten viruses associated with bovine respiratory disease detected in samples collected at 14 days on feed (DOF) between fall-placed calves (FPC) and yearlings (YRL) in western Canadian feedlots.

Virus	FPC 14 DOF samples: (*n* = 260)		YRL 14 DOF samples: (*n* = 120)		14 DOF samples: YRL compared to FPC		
	Prevalence	95% CI	Prevalence	95% CI	Odds ratio	95% CI	*p*-value
Viruses with cutoff for positive status based on BLCM (67)							
IDV	51%	36–67%	33%	5.3–61%	0.48	0.12–1.95	0.30
BRSV	26%	9.4–42%	60%	26–94%	4.32	0.83–22.5	0.08
BPIV-3	12%	2.9–20%	39%	13–65%	4.94	1.26–19.3	**0.02**
BCoV	10%	4.2–17%	18%	0–43%	1.83	0.28–11.8	0.53
BoHV-1	2.7%	0–5.9%	0%	0–3.0%	0.22	0–1.49	0.14
Viruses for which there are no BLCM-informed cutoffs – Sample positive if ≥ 1 read							
BRBV	38%	26–50%	27%	1.3–52%	0.59	0.15–2.38	0.46
UCPV1	17%	10–24%	7.5%	1.8–13%	0.39	0.15–1.01	0.05
BVDV-2	1.5%	0–3.8%	17%	1.3–32%	12.8	1.97–83.3	**0.01**
BAdV-3	0.4%	0–1.1%	1.7%	0–3.6%	4.39	0.45–42.7	0.20
BVDV-1	0%	0–1.4%	3.3%	0–7.5%	11.7	1.45-∞	**0.02**

The cutoffs used to determine virus-positive samples were based on ≥ 1 read for all viruses except BPIV-3 (≥ 5 reads) and BCoV (≥ 30 reads).

FPC: fall-placed calves; YRL: yearlings; BCoV: bovine coronavirus; BAdV-3: bovine adenovirus 3; BoHV-1: bovine herpesvirus 1; BPIV-3: bovine parainfluenza virus 3; BRBV: bovine rhinitis B virus; BRSV: bovine respiratory syncytial virus; BVDV-1: bovine viral diarrhea virus 1; BVDV-2: bovine viral diarrhea virus 2; IDV: influenza D virus; UCPV1: ungulate copiparvovirus 1; CI: confidence interval; BLCM: Bayesian latent class models. Comparable tables were produced based on reads not adjusted for water controls (Supplementary Table S2D) and reads adjusted for the medians of the water controls (Supplementary Table S3D). The bold values indicate associations that are statistically significant.

### 3.8 Sensitivity analysis

Comparable analyses to those in Tables 2–5 were conducted without adjustment for water controls or using the median (rather than the mean) read counts for the water controls (Supplementary Tables S2A–D, S3A–D). Using the mean read counts from the water controls for adjustment yielded more conservative outcomes (i.e., fewer samples classified as positive) compared to the no adjustment and slightly more conservative outcomes than when adjusting for the median of the water controls. Significant differences between viruses detected at 14 DOF and at arrival processing and between FPC and YRL were very similar regardless of what adjustment for water controls was used.

### 3.9 Detection of BRD bacterial pathogens and ARGs in the viral metagenomic sequencing data

The same metagenomic dataset used for viral analysis identified 145,005 reads classified as bacterial species of interest for BRD. Based on detection of a single read, *Histophilus somni*, *Mannheimia haemolytica*, and *Pasteurella multocida* were most abundant (Table 6).

**Table 6:** Read characteristics of select respiratory bacteria detected in nasal samples collected at arrival processing from fall-placed calves (*n* = 520) and yearlings (*n* = 240) in western Canadian feedlots.

Summary of bacterial read data	Total reads from all samples	Proportion of samples ≥ 1 read detected	Median (maximum) of sample median read lengths (bp)*
*Mannheimia haemolytica*	108,298	59%	789 (2481)
*Pasteurella multocida*	21,687	41%	760 (3202)
*Histophilus somni*	7,806	67%	224 (1248)
*Bibersteinia trehalosi*	7,006	16%	508 (3652)
*Mycoplasmopsis bovis*	208	5%	571 (2240)

* Median reported for samples where the virus was detected in at least one read. Individual read lengths were summarized for each sample and then sample medians were summarized across all samples.

However, when thresholds for detection based on read count cutoffs informed by receiver operating characteristic (ROC) curves and BLCM were used (67), the overall frequency of detection for bacteria of interest was as follows: *M. haemolytica* (43% ≥ 14 reads), *H. somni* (28% ≥ 8 reads), *P. multocida* (17% ≥ 10 reads), *Mycoplasmopsis bovis* (formerly known as *Mycoplasma bovis*) (16% ≥ 1 read), and *Bibersteinia trehalosi* (4.6% ≥ 1 read).

Based on the BLCM-based thresholds for detection, *M. haemolytica* and *H. somni* were the most prevalent bacteria detected in FPC arrival samples, followed by *P. multocida* (Table 7). The frequency of detection of *M. haemolytica* was higher in 14 DOF samples than in arrival samples (*p =* 0.02); however, the frequency of detection of *P. multocida* was lower in the 14 DOF samples (*p =* 0.03). While *M. bovis* was only identified in 2.7% of arrival samples, the prevalence increased to 37% in 14 DOF samples (*p <* 0.001). Within the FPC samples, no significant changes in the prevalences of *H. somni* or *B. trehalosi* between arrival and 14 DOF were noted.

**Table 7:** Differences in prevalence of bacterial pathogens associated with bovine respiratory disease using a viral metagenomics sequencing protocol from 520 nasal swabs collected from fall-placed calves (FPC) sampled at arrival processing and at 14 days on feed (DOF).

Fall-placed calves											
Bacteria	Sample time 1: arrival processing (*n* = 260)				Sample time 2: 14 DOF (*n* = 260)				14 DOF compared to arrival		
	No. pos. samples	Prevalence	95% CI	No. reads	No. pos. samples	Prevalence	95% CI	No. reads	Odds ratio	95% CI	*p*-value
*Mannheimia haemolytica*	92	35%	23–51%	15,945	137	53%	36–69%	55,864	2.03	1.11–3.71	**0.02**
*Histophilus somni*	92	35%	24–49%	3,313	88	34%	23–47%	3,672	0.93	0.61–1.43	0.75
*Pasteurella multocida*	60	23%	16–32%	9,728	30	12%	7–19%	6,929	0.43	0.21–0.92	**0.03**
*Mycoplasmopsis bovis*	7	2.7%	0.9–8.2%	17	95	37%	26–49%	6,832	20.8	7.60–57.0	**<0.001**
*Bibersteinia trehalosi*	12	4.6%	2.4–8.8%	79	10	3.8%	1.6–9.0%	21	0.83	0.24–2.86	0.76

The cutoffs used to determine bacteria-positive samples were based on BLCM results for *M. haemolytica* (≥ 14 reads), *P. multocida* (≥ 10 reads), *H. somni* (≥ 8 reads), and *M. bovis* (≥ 1 read). A cutoff of ≥ 1 read was used for *B. trehalosi*.

FPC: fall-placed calves; DOF: days-on-feed; CI: confidence interval, NO: number; POS: positive. The bold values indicate associations that are statistically significant.

The most prevalent bacterial species identified in YRL samples collected at both arrival processing and 14 DOF was *M. haemolytica* followed by *P. multocida*, *H. somni*, *M. bovis*, and *B. trehalosi* (Table 8). Within the YRL samples, no significant differences in the prevalences of any of these bacteria between arrival and 14 DOF were noted.

**Table 8:** Differences in prevalence of bacterial pathogens associated with bovine respiratory disease using a viral metagenomics sequencing protocol from 240 nasal swabs collected from yearlings (YRL) sampled at arrival processing and at 14 days on feed (DOF).

Yearlings											
Bacteria	Sample time 1: arrival processing (*n* = 120)				Sample time 2: 14 DOF (*n* = 120)				14 DOF compared to arrival		
	No. pos. samples	Prevalence	95% CI	No. of reads	No. pos. samples	Prevalence	95% CI	No. of reads	Odds ratio	95% CI	*p*-value
*Mannheimia haemolytica*	40	33%	33–72%	12,194	55	46%	60–92%	24,295	1.69	0.95–3.00	0.07
*Pasteurella multocida*	19	16%	12–62%	1,174	23	19%	26–67%	3,856	1.26	0.39–4.04	0.70
*Histophilus somni*	13	11%	42–77%	407	17	14%	48–77%	414	1.36	0.50–3.71	0.55
*Mycoplasmopsis bovis*	6	5.0%	1.4–16%	33	15	13%	3.0–40%	124	2.71	0.70–10.5	0.15
*Bibersteinia trehalosi*	4	3.3%	0.8–13%	51	9	7.5%	4.2–13%	57	2.35	0.79–7.02	0.13

The cutoffs used to determine bacteria-positive samples were based on BLCM results for *M. haemolytica* (≥ 14 reads), *P. multocida* (≥ 10 reads), *H. somni* (≥ 8 reads), and *M. bovis* (≥ 1 read). A cutoff of ≥ 1 read was used for *B. trehalosi*.

YRL: yearlings; DOF: days-on-feed; CI: confidence interval, NO: number; POS: positive.

Other respiratory bacteria of interest were also detected in these samples. For example, at least five reads of *Mesomycoplasma dispar* were detected in 27% (71/260) of FPC arrival samples and 40% (105/260) of 14 DOF samples. Similarly, at least five reads of *M. dispar* were detected in 28% (34/120) of YRL arrival samples and 38% (46/120) of 14 DOF samples. By comparison, 11% of all samples (83/760) had at least five reads for *M. bovis*: 1% of FPC arrival samples (2/260), 30% of FPC 14 DOF samples (77/260), and 2% (2/120) of both YRL arrival and 14 DOF samples.

We used a higher threshold (five reads vs. one read) for bacteria not targeted in the original search before reporting their presence in a sample. At least five reads of bacteria of potential interest were also identified in this sequence data in at least five of the 760 samples: *Moraxella bovoculi* (251 (33%)), *Moraxella bovis* (158 (21%)), *Mycoplasmopsis bovirhinis* (77 (10%)), *Mannheimia bovis* (21 (2.8%)), *Metamycoplasma alkalescens* (17 (2.2%)), *Mycoplasmopsis bovigenitalium* (6 (0.8%)), and *Ureaplasma diversum* (5 (0.7%)).

ARGs were also identified in the metagenomics dataset utilized for viral analyses (Table 9). Specifically, 100 reads corresponding to 33 unique ARGs were detected in 48 (6.3%) of 760 nasal samples. The prevalence of at least one ARG was 3.3% in FPC arrival samples, 8.3% in FPC 14 DOF samples, 3.3% in YRL arrival samples, and 12.5% in YRL 14 DOF samples. The prevalence was significantly higher in the 14 DOF samples than the arrival samples for both FPC (*p* = 0.03) and YRL (*p* = 0.01). The average sequence coverage was 94.7%, and the average sequence identity was 93.3% for ARGs compared to similar sequences in the NCBI and CARD databases. The *lnu*(*C*) gene, which confers resistance to lincosamides, was the most prevalent ARG detected (1.8%), followed by *aph(3′)-Ia* (0.9%), which confers aminoglycoside resistance (Table 9).

**Table 9:** Detection of antimicrobial resistance genes (ARGs) using a viral metagenomics sequencing protocol from 760 nasal swabs collected from fall-placed calves (FPC) and yearlings (YRL).

ARGs	# of ARGs detected in FPC nasal samples			# of ARGs detected in YRL nasal samples			Total # of ARGs detected	# of all samples ≥ 1 read (%)	Resistance type
	Arrival	14 DOF	Total FPC	Arrival	14 DOF	Total YRL			
*aad9*		1	1		1	1	2	2 (0.3%)	aminoglycoside
*aadA1*	1		1				1	1 (0.1%)	aminoglycoside
*aadA27*					2	2	2	2 (0.3%)	aminoglycoside
*aadD1*		1	1				1	1 (0.1%)	aminoglycoside
*ANT(3″)-IIa*	1		1				1	1 (0.1%)	aminoglycoside
*ANT(4′)-Ia*		3	3				3	3 (0.4%)	aminoglycoside
*APH(3′)-Ia*		5	5	6	1	7	12	7 (0.9%)	aminoglycoside
*aph(3′)-Id*		1	1				1	1 (0.1%)	aminoglycoside
*APH(6)-Id*		1	1				1	1 (0.1%)	aminoglycoside
*bacA*					1	1	1	1 (0.1%)	bacitracin
*CfxA2*					4	4	4	2 (0.3%)	cephamycin
*dfrA44*				2		2	2	1 (0.1%)	trimethoprim
*dfrD*		1	1				1	1 (0.1%)	diaminopyrimidine
*ErmC*		1	1		1	1	2	2 (0.3%)	lincosamide; macrolide; streptogramin; streptogramin_A; streptogramin_B
*ErmG*	1		1				1	1 (0.1%)	lincosamide; macrolide; streptogramin; streptogramin_A; streptogramin_B
*evgA*	1		1		10	10	11	2 (0.3%)	fluoroquinolone; macrolide; penam; tetracycline
*gadW*					2	2	2	2 (0.3%)	fluoroquinolone; macrolide; penam
*lnuC*	1	22	23	1	4	5	28	14 (1.8%)	lincosamide
*mdtM*		1	1				1	1 (0.1%)	disinfecting_agents_and_antiseptics; fluoroquinolone; lincosamide; nucleoside; phenicol
*mel*		2	2				2	1 (0.1%)	macrolide; streptogramin
*OmpA*		1	1				1	1 (0.1%)	peptide
*patB*		1	1		1	1	2	2 (0.3%)	fluoroquinolone
*PC1_beta-lactamase_(blaZ)*					3	3	3	1 (0.1%)	penam
*qacEdelta1*	1		1				1	1 (0.1%)	disinfecting_agents_and_antiseptics
*RlmA(II)*				1		1	1	1 (0.1%)	lincosamide; macrolide
*SAT-2*	1		1				1	1 (0.1%)	nucleoside
*SAT-4*		1	1		1	1	2	2 (0.3%)	nucleoside
*sul2*		1	1		1	1	2	2 (0.3%)	sulfonamide
*tet (40)*		1	1		1	1	2	2 (0.3%)	tetracycline
*tet(H)*		1	1				1	1 (0.1%)	tetracycline
*tet(Q)*		1	1				1	1 (0.1%)	tetracycline
*tet(W)*	3		3				3	2 (0.3%)	tetracycline
*YajC*					1	1	1	1 (0.1%)	cephalosporin; disinfecting_agents_and antiseptics; fluoroquinolone; glycopeptide; glycylcycline; oxazolidinone; penam; phenicol; rifamycin; tetracycline
Total	10	46	56	10	34	44	100	48 (6.3%)	

## 4 Discussion

A single metagenomic sequencing protocol was used to detect a wide variety of respiratory RNA viruses, DNA viruses, and bacteria in nasal swabs collected from both FPC and YRL in the early feeding period. A nanopore-based protocol was adapted to efficiently process a large number of samples collected by private veterinarians from commercial feedlots in collaboration with a national surveillance initiative. These results demonstrate the value of metagenomic sequencing for detecting respiratory viruses, which are routinely targeted for testing, as well as previously documented respiratory viruses for which routine commercial laboratory tests are unavailable (70).

To the best of our knowledge, this is the first study to examine the prevalence of respiratory viruses in feedlot cattle during the initial feeding period. These results offer insights into the dynamics of recognized BRD viral pathogens and other viruses early in their time in the feedlot. While the prevalences of various respiratory viruses in feedlot cattle have been reported (24, 32, 38, 40), the current study provides new insights within sampled pens of two age groups of cattle (younger FPC and older YRL) at two time points during the initial few weeks of the feeding period when BRD risk is high.

Most metagenomic studies on BRD viruses in cattle have concentrated on the period when cattle are undergoing treatment for BRD (38–40). In contrast, only one prior metagenomic study explored these viruses in feedlot cattle at arrival processing, before the onset of BRD, and then assessed the link between the identified viruses and BRD development within 40 DOF (24). The present study further builds on work by Zhang et al. (24). Although our investigation and the study by Zhang et al. (24) both identified over 20 different viruses in respiratory samples collected at feedlot arrival, the collection methods differed. Additionally, our research employed short nasal swabs whereas Zhang et al. (24) used deep nasopharyngeal swabs. The present study also collected nasal samples from cattle at 14 DOF from the same pens sampled at arrival processing.

We identified 21 viruses from 12 virus families in nasal swabs collected from FPC and YRL as part of routine surveillance sampling in western Canadian feedlots, including the viruses currently included in commercial respiratory vaccines. In particular, the prevalences of BPIV-3 and BRSV were higher in arrival samples than other viruses included in five-way respiratory vaccines. This finding aligns with other metagenomic studies in feedlot cattle, with detection of BPIV-3 and BRSV in samples from both acute BRD cases and asymptomatic controls, as well as in cattle sampled at arrival processing (24, 32, 38).

Most samples collected from FPC and YRL tested positive for at least one respiratory virus. This finding is consistent with a previous metagenomic analysis in western Canadian feedlots (24) that reported 75% of sampled cattle had at least one respiratory virus detected at arrival processing. Results from other metagenomic investigations vary. For instance, a case–control study using shorter-read Illumina MiSeq in Australia by Ambrose et al. (40) found only 33% of feedlot cattle with BRD tested positive for at least one virus. In contrast, research on feedlot steers with acute BRD and their asymptomatic pen-mates in the United States and Mexico (38) revealed 80% of nasal swabs from the animals were positive for at least one viral agent.

The observed differences in the frequency of viruses detected in previous studies could be attributed to variations in sampling sites within the respiratory tract, the type of assay used for detection, bioinformatics approaches, and study designs. Factors such as differences in animal age, BRD disease status, management including vaccine use, and geographic location may also play a role (32, 38, 71, 72). While Mitra et al. (38) and Zhang et al. (24) employed metagenomic assessments for virus detection, Ambrose et al. (40) supplemented metagenomic assessments with real-time multiplex PCR results. Overall, the high frequency of virus detection across these studies aligns with the observation that some of these respiratory viruses are considered endemic in cattle (73–75).

The present study identified many viruses that are currently included in commercial BRD vaccines. Most FPC were likely vaccinated at arrival processing with an injectable modified live virus (MLV) vaccine against BoHV-1, BVDV-1, BVDV-2, BRSV, and BPIV-3 following typical recommendations of the consulting feedlot veterinary practices (76, 77). However, specific details about the type of vaccine and the route of administration for either FPC or YRL were not disclosed by the feedlots involved in this study, but these factors could have influenced the outcomes of the study (78). Given the unknown arrival vaccination protocols, some differences observed between arrival processing and 14 DOF might be attributed to the detection of vaccine virus rather than changes in the prevalence of wild-type virus. Recent publications from western Canada (21, 22, 79, 80) suggest many FPC entering the feedlot could have originated from herds that administered combination BVDV, BoHV-1, BPIV-3, and BRSV vaccines to the cow-herd and often at least once to calves before weaning. The metagenomic and bioinformatic protocols focusing on individual reads did not distinguish between field versus vaccine virus.

The relatively low prevalences or lack of detection of BVDV-1, BVDV-2, and BoHV-1 in the early feeding period of this study population indicated a low level of exposure to these viruses, possibly due to the effectiveness of commercial vaccines and vaccination programs in the source herds. A low prevalence of BVDV-1, BVDV-2, and BoHV-1 has been reported in deep nasopharyngeal samples collected from feedlot cattle (24, 32, 38), supporting the premise that current respiratory viral vaccines should be relatively effective in reducing transmission of viruses among herds (81, 82). BoHV-1 was found solely in samples collected from FPC at approximately 2 weeks on feed. While we lack data to confirm latent BoHV-1 infections in the calves, stressors such as transport to feedlots or corticosteroid treatment can cause reactivation and shedding of latent BoHV-1 (83).

In contrast, the slightly higher prevalences of BRSV and BPIV-3 in samples collected at arrival processing may suggest the available respiratory vaccines are not equally effective against all circulating strains of these viruses (84, 85) or reflect the absence of prime-boosting strategies (86). The BRSV vaccine is considered a core vaccine for beef herds by both the American Association of Bovine Practitioners and a recent vaccine expert committee review in western Canada (79, 87). The detection of BRSV at arrival processing could be linked to the vaccine, highlighting either the relative effectiveness of the available vaccines or the current administration practices (86). This is particularly pertinent to the need to improve the application of these vaccines in nursing calves before weaning and feedlot entry (21).

The observed increase in BRSV prevalence after 2 weeks may be attributed to the mingling of animals from various sources, the circulation of the native virus within pens over time, or the potential for shedding virus from modified-live vaccines typically administered at arrival processing. Calves can shed BRSV for up to 4 weeks following natural exposure (88), but typically less than 2 weeks after receiving subcutaneous (SC) MLV vaccines (89). However, in the relatively unlikely situation of an intranasal (IN) MLV vaccine used at feedlot arrival processing, shedding might extend to 28 days post-vaccination (78). Unfortunately, no BRD management data were available for this cattle cohort, making it impossible to determine if the shedding animals were at a heightened risk of disease. Nonetheless, another study found calves with BRD were more likely to test positive for BRSV compared to healthy controls (32).

BRSV has been detected in other metagenomic studies of BRD in cattle, appearing in both sick animals and asymptomatic controls (24, 32, 38), and is reported to be endemic worldwide (74). The severity of BRSV infection is influenced by the calf’s immunity, with higher antibody levels linked to reduced morbidity and mortality (90). Vaccinating calves within their first month of life decreases morbidity and mortality from BRSV infection (86, 91). Nevertheless, the success of this approach has been inconsistent due to the interference of colostrum-derived serum antibodies, which hinder the vaccine-induced response (86).

The similarity in the trend of increasing prevalence might not be unexpected for BPIV-3 and BRSV. These viruses were classified, until 2016, under the same family, sharing numerous characteristics and often being targeted together in combination vaccines due to their similar pathobiological and epidemiological features (24, 32, 38). The higher prevalences of these two viruses in 14 DOF samples might be associated with the shedding of wild-type virus from natural infection. While the calves in this study were likely vaccinated, the immune response might not have been adequate to reduce shedding post-infection. A recent study found that, for both viruses, shedding was lower after challenge if calves were primed by an MLV vaccine and boosted with an inactivated virus vaccine when compared to the current industry standard of MLV priming and boosting (81). This could indicate current standard/common immunization protocols do not result in effective immunity.

BPIV-3 is an RNA virus in the *Respirovirus* genus within the *Paramyxoviridae* family (72). Although BPIV-3 has been reported to cause broncho-interstitial pneumonia (92), its clinical importance in feedlot cattle remains uncertain. In North America, both killed/inactivated and MLV BPIV-3 vaccines are used (20). Molecular tests, such as real-time PCR and virus genome sequencing, have identified three distinct types of BPIV-3—A, B, and C—each with antigenic variations (85, 93). Commercial MLV vaccines for BPIV-3 typically include the A type. BPIV-3 strains from cattle that have been administered MLV vaccines and later diagnosed with BRD could exhibit respiratory tract strains that differ from the MLV vaccine strains (20). Future metagenomic studies could explore the potential for typing BRD virus strains such as BPIV-3.

Among respiratory viruses with positive cutoffs established by BLCM (67), BCoV was the most frequently detected virus in FPC arrival samples with a significantly lower prevalence in samples collected 2 weeks later. The high prevalence of BCoV observed in arrival samples is consistent with a previous metagenomics study that collected deep nasopharyngeal swabs from western Canadian feedlot cattle at arrival processing (24). Mitra et al. (38) also found BCoV to be one of the most commonly identified viruses in their metagenomic sequencing study of nasal swab samples from Mexican- and US-sourced feedlot cattle with acute BRD and asymptomatic pen-mates. BCoV is a member of the family *Coronaviridae*, Subgroup 2a, genus *Betacoronavirus* and the order *Nidovirales*. Genome sequencing and characterization of the BCoV spike protein gene have identified two clades, BCoV1 and BCoV2, that display antigenic differences (94). BCoV has been isolated from both healthy and sick calves and is a primary cause of calf diarrhea and winter dysentery in adult cattle (94). Additionally, researchers have reported a link between BCoV and BRD (95, 96). More recently, another metagenomics study that sampled western Canadian feedlot cattle, with and without BRD, reported BCoV was significantly associated with BRD (32). However, no studies have demonstrated measurable gross and microscopic lesions of pneumonia in the lungs when susceptible calves are naturally or experimentally challenged with BCoV (20, 96). Additional research is required to determine the pathogenicity of BCoV and if new respiratory vaccines are required for its control.

Our detection of IDV in this study is consistent with previous reports from metagenomics studies that identified IDV in respiratory samples collected at arrival processing (24) from both sick and asymptomatic cattle (32, 38–40). The identification of IDV sequences in both sick and asymptomatic cattle can be attributed to cattle being natural reservoirs, making them more likely to carry the virus without displaying clinical signs of disease. IDV, which belongs to the *Orthomyxoviridae* family, genus *Deltainfluenzavirus*, is increasingly detected around the world and has been reported to be efficiently transmitted among cattle through direct contact (20, 97). While IDV generally causes mild to moderate upper respiratory disease in cattle, reports regarding its association with BRD are conflicting (24, 38). Changes in the upper respiratory tract due to IDV infection could intensify the effects of coinfecting pathogens (6, 24, 40).

Previous metagenomic studies have reported the detection of BRBV in respiratory samples of cattle with and without BRD (32, 38–40), with a significant association with BRD described in one of the previous reports (32). Our current findings further corroborate that BRBV is common among western Canadian cattle, irrespective of the sampling period. Although BRBV was the most frequently detected virus in FPC and YRL arrival samples, overestimation of the frequency of detection is possible without an established BLCM threshold for BRBV. For previously discussed BRD viruses, the assessment of presence or absence in the sample after adjustment for water controls was based on a cutoff established through comparison to qPCR results with BLCM. As no such qPCR data were available for BRBV and the remaining viruses, all assessments of prevalence were limited to classifying the samples based on the detection of at least one read, and so the prevalence could be overestimated relative to the more commonly studied viruses described in the BLCM (67).

Bovine adenovirus type 3 (BAdV3) was identified although with a very low prevalence. BAdV3 has been detected in metagenomic studies of cattle (24, 32, 38) and linked to a range of diseases, including conjunctivitis, pneumonia, diarrhea, and polyarthritis (98). In addition, Ng et al. (39) found BAdV3 was significantly associated with BRD.

Cattle harbor a variety of parvoviruses, including ungulate bocaparvoviruses (UBPV), bovine adeno-associated virus (BAAV), ungulate erythroparvoviruses (UEPV), ungulate tetraparvoviruses (UTPV), and ungulate copiparvoviruses (UCPV) (99). The six species/variants detected in the current study, UBPV1, UBPV6, UCPV1, UCPV5, UEPV1, and UTPV1, have been reported in other metagenomic studies (24, 32, 38–40, 100, 101). The number of identified animal parvoviruses has increased substantially due to the application of high-throughput metagenomic sequencing (42). While parvoviruses are widely acknowledged as important pathogens in various mammalian species, few published studies have explored their clinical significance in cattle. Bovine parvoviruses have, however, been linked to both respiratory and gastrointestinal diseases (102).

Other viruses detected included bovine nidovirus 1, an emerging pathogen reported in cattle in the United States, Canada, and Australia (32, 40, 103). Bovine polyomavirus 2 is commonly regarded as a contaminant in tissue culture serum but has more recently been linked to nonsuppurative encephalitis in cattle (104). Finally, bovine papillomavirus is a very common virus that causes cutaneous papillomas in cattle (105).

This study is the first to simultaneously identify and report BRD viruses, BRD bacteria, and bacteria-associated ARGs from metagenomic analysis using a single metagenomic protocol. The current range of processes, including qPCR, culture, and susceptibility testing used by diagnostic laboratories to generate both viral and bacterial data from respiratory samples is labor-intensive, expensive, time-consuming, and limited to detecting known and targeted pathogens. Here, a large proportion of non-viral reads remained even after removal of host sequences during bioinformatic preprocessing, suggesting the potential for previously unexplored useful sequence data. From the viral metagenomics data, results were generated for four *Pasteurellaceae* bacteria of interest in addition to *Mycoplasmopsis bovis*. Bayesian latent class models applied to these data showed the clinical sensitivities for three species (*M. haemolytica*, *P. multocida*, and *H. somni*) were comparable to or exceeded culture under field conditions and the sensitivity was comparable to qPCR for *Mycoplasmopsis bovis* (67).

The present analyses expand upon that work to explore the larger range of bacteria and viruses detected in these viral metagenomics protocol data. While *Bibersteinia trehalosi* was not cultured as part of the CFAASP program (47) that provided access to these samples, the organism was readily detected in the metagenomics data with differences in FPC and YRL noted over the period considered in the present study. As another example, the summary script contained within the bioinformatic pipeline generated in the analysis contained read information for several of the *Mycoplasmataceae* family, with *Mesomycoplasma dispar*, formerly *Mycoplasma dispar*, being more frequently identified than *Mycoplasmopsis bovis*. Other routinely detected respiratory bacteria of interest included *Moraxella bovoculi*, *Moraxella bovis*, *Mycoplasmopsis bovirhinis*, and *Mannheimia bovis*. The enhanced detection capability demonstrated by this metagenomic approach enables more efficient and comprehensive identification of pathogens of interest and the potential for improved understanding and treatment of BRD. The ability to balance processing time, input costs, and the generation of on-target data for a range of pathogens makes this approach particularly valuable and immensely beneficial for practical field applications and the industry (45).

ARGs were detected by metagenomics sequencing in only a few (6.3%) of the 760 nasal samples collected in the early feeding period. Many of the ARGs detected were associated with resistance to drugs used in the cattle industry (3), including macrolides (*ErmC, ErmG, evgA, mel, RlmA(II)*), phenicols (*mdtM, YajC*), tetracyclines (*tet (40), tet(H), tet(Q), tet(W)*), fluoroquinolones (*patB, evgA*), and sulfonamides (*sul2*). Some ARGs associated with resistance to antimicrobials not used in the cattle industry were also detected: lincosamide (*lnuC*) and aminoglycosides (*aadA27, ANT(3″)-IIa, ANT(4′)-Ia, ANT(3′)-Ia, APH(6)-Id*). Interestingly, the two most prevalent ARGs detected by metagenomics sequencing (*lnu(C)* and *aph(3′)-Ia*) are associated with resistance to antimicrobial classes not used in the Canadian cattle industry. The detected ARGs are consistent with those documented in previous research, including recent proof-of-concept metagenomic studies in feedlot cattle (44, 45) and DNA-based surveys of the bovine respiratory tract (106–108) and other sites (109). The number of ARGs detected increased in 14 DOF samples compared to arrival samples, potentially due to selection associated with antimicrobial metaphylaxis at arrival processing and the mixing of animals from different herds, which facilitated contagious transmission of bacteria-associated ARGs (110). Although specific information on the type of metaphylaxis used was unavailable due to data anonymity and confidentiality, reports from veterinarians and these feedlots suggest tulathromycin was likely common in the FPC, while oxytetracycline was more prevalent among YRL (3). This is consistent with the detection of ARGs to macrolides (*ErmC*: 0 at arrival to 2 reads at 14 DOF; *mel*: 0 at arrival to 2 reads at 14 DOF) in FPC and oxytetracycline (*evgA*: 0 at arrival to 10 reads at 14 DOF) in YRL during the early feeding period.

While the preparation protocol designed to detect both DNA and RNA viruses successfully identified BRD-associated bacteria, the bacterial reads were substantially shorter on average and fewer in number than reported in a study using a protocol optimized for bacterial metagenomics (45). The shorter bacterial reads from the current protocol could have resulted from the sample processing and cDNA amplification with PCR required for simultaneous RNA virus detection. Herman et al. (45) used a preparation method that included non-specific culture-based enrichment of specimens and no processing steps that reduced the read length, leading to more and longer bacterial sequence reads and a higher proportion of BRD bacterial reads containing one or more ARGs. Similar to the current study, a previous paper not using the enrichment step also found ARGs but did not focus on specific bacterial species of interest (44).

The existing literature based on culture and PCR methods has reported a low initial presence of *H. somni* in feedlot cattle, followed by an increase in prevalence after arrival (111–113). In contrast, we observed similar prevalences of *H. somni* and *M. haemolytica* in arrival samples in FPC. However, no remarkable changes in *H. somni* positive samples or number of reads were detected in 14 DOF samples. Notwithstanding our findings of a higher *H. somni* prevalence than expected in FPC (114), most samples exhibited low absolute read counts when compared to *M. haemolytica*, suggesting low abundance. This phenomenon could be one of the reasons why *H. somni* are challenging to culture from samples obtained from cattle upon arrival (113).

The present study leveraged the existing CIPARS national surveillance program to provide samples from a large number of commercial feedlots. The choice to sample 20 calves per pen from each feedlot was limited by the collaboration with the surveillance project. However, previous work by this research group has shown that 20 calves does provide sufficient information to differentiate pens with low, moderate and high prevalence (112).

A major advancement in this study was the use of the PromethION platform which empowered the simultaneous processing of hundreds of samples (115). The advancements in throughput dramatically impact the feasibility of implementing metagenomics diagnostics in high-volume, real-world settings such as feedlots.

One potential limitation of the current study was the use of nasal swabs versus deep nasal pharyngeal swabs to collect respiratory mucosa samples. Nasal swabs were selected due to their practicality, non-invasiveness, ease of transport, and cost-effectiveness in field conditions. McDaneld et al. (116) found both nasal swabs and deep nasopharyngeal swabs yielded similar results in detecting bacterial populations in healthy and BRD-affected cattle. However, Zhang et al. (32) found a poor correlation between the detection of various viruses in deep nasopharyngeal swabs and tracheal washes from BRD-affected cattle. Consequently, all the viruses infecting the calves may not have been identified given the type of sample used, particularly if the virus affects a different part of the respiratory tract than the sampled nasal cavity. However, it is important to note that these samples were taken from randomly selected calves from feedlot pens during arrival and 14 DOF processing. As a result, most calves would not be exhibiting signs of disease when sampled. In this instance, the focus is on detection of viruses circulating within a pen of cattle as opposed to diagnosing viruses contributing to acute BRD (117).

Another factor that could have influenced the sensitivity of detection is the nucleic acid extraction kit used. Different viral DNA/RNA extraction kits used in metagenomics analysis have varying extraction efficiencies for different viruses, which can ultimately affect the sensitivity of the results and outcome of the assay (118). Several studies have emphasized the importance of choosing the appropriate extraction kit to minimize potential biases and improve the accuracy of the results (71, 118, 119). The high concentration of host DNA reads observed in this study suggests the need for an alternative to the QIAamp MinElute Virus Spin Kit for removing host DNA (118). Furthermore, the kit’s performance in metagenomic analysis has not been as robust as some others, particularly in terms of host DNA removal and viral read proportion (118).

One limitation of bioinformatics analysis is the scarcity of reference genome data for bovine respiratory viruses, which can result in false negatives, especially at the strain level (120). Additionally, base-calling error rates can amplify this problem. Nevertheless, with the production version used in this analysis (R10.4.1 chemistry), nanopore sequencing can achieve more than 99.99% raw read accuracy (121, 122).

While PCR remains the most commonly used method for detecting BRD viruses in veterinary clinical laboratories, the increasing affordability of metagenomic sequencing has expanded the ability to identify a broader range of microbial pathogens in clinical samples. Nanopore sequencing can serve as a viable alternative to PCR, as it offers relatively low-cost, rapid, real-time data streaming from next-generation long-read sequencing, providing immediate access to results. Additionally, a laboratory test capable of detecting relevant viruses and bacteria, including some ARGs, could reduce both management costs and turnaround time in the diagnosis of BRD.

## 5 Conclusion

This study is the first to employ metagenomic sequencing to concurrently detect viruses, bacteria, and bacteria-associated antimicrobial resistance genes (ARGs) from nasal samples of feedlot cattle, using a protocol specifically designed to enhance virus detection. This further underscores the value of nanopore metagenomics sequencing as a comprehensive ‘one test for all’ method for identifying both viral and bacterial pathogens linked to bovine respiratory disease (BRD). The metagenomic sequencing data from this research enrich the understanding of the diversity, prevalence, and dynamics of both well-known and emerging viruses potentially associated with clinical BRD in the early feeding period. The study also highlights potential shortcomings in current commercial respiratory vaccines or vaccination protocols against existing field strains of BRSV and BCoV.

Additional studies are required to establish standardized metagenomic techniques for regular clinical diagnoses of BRD, examine the occurrence of these and other viruses in asymptomatic cattle, and clarify the connection between microbial dysbiosis and illness. Moreover, enhancing knowledge regarding viruses that play a role in BRD in cattle will aid in the creation of effective control and prevention methods. This encompasses the development and implementation of novel laboratory tools to detect pathogens and vaccines, as well as the enhancement of existing ones, to help reduce the impact of BRD on the global beef industry.

## Data Availability

The genomic data used in this study have been deposited in the Sequence Reach Archive (SRA) within BioProject ID: PRJNA1374179. Custom scripts can be accessed at: https://github.com/coadunate/ASSETS_2 (accessed on 26 October 2025).

## References

[1] Loneragan GH Dargatz DA Morley PS Smith MA. Trends in mortality ratios among cattle in US feedlots. J Am Vet Med Assoc. (2001) 219:1122–7. doi: 10.2460/javma.2001.219.1122, 11700712

[2] Blakebrough-Hall C Hick P Mahony TJ Gonzalez LA. Factors associated with bovine respiratory disease case fatality in feedlot cattle. J Anim Sci. (2022) 100:skab361. doi: 10.1093/jas/skab361, 34894141 PMC8796815

[3] Brault SA Hannon SJ Gow SP Warr BN Withell J Song J . Antimicrobial use on 36 beef feedlots in western Canada: 2008-2012. Front Vet Sci. (2019) 6:329. doi: 10.3389/fvets.2019.00329, 31681801 PMC6811515

[4] Waldner CL Parker S Gow S Wilson DJ Campbell JR. Antimicrobial usage in western Canadian cow-calf herds. Can Vet J. (2019) 60:255–67. 30872848 PMC6380250

[5] Gaudino M Nagamine B Ducatez MF Meyer G. Understanding the mechanisms of viral and bacterial coinfections in bovine respiratory disease: a comprehensive literature review of experimental evidence. Vet Res. (2022) 53:70. doi: 10.1186/s13567-022-01086-1, 36068558 PMC9449274

[6] Saegerman C Gaudino M Savard C Broes A Ariel O Meyer G . Influenza D virus in respiratory disease in Canadian, province of Quebec, cattle: relative importance and evidence of new reassortment between different clades. Transbound Emerg Dis. (2022) 69:1227–45. doi: 10.1111/tbed.14085, 33764631

[7] Timsit E Christensen H Bareille N Seegers H Bisgaard M Assie S. Transmission dynamics of *Mannheimia haemolytica* in newly-received beef bulls at fattening operations. Vet Microbiol. (2013) 161:295–304. doi: 10.1016/j.vetmic.2012.07.044, 22901531

[8] Pardon B Buczinski S. Bovine respiratory disease diagnosis: what progress has been made in infectious diagnosis? Vet Clin North Am Food Anim Pract. (2020) 36:425–44. doi: 10.1016/j.cvfa.2020.03.005, 32451034 PMC7244442

[9] Gupta RS Oren A. Necessity and rationale for the proposed name changes in the classification of *Mollicutes* species. Reply to: 'Recommended rejection of the names *Malacoplasma* gen. nov., *Mesomycoplasma* gen. nov., *Metamycoplasma* gen. nov., *Metamycoplasmataceae* fam. nov., *Mycoplasmoidaceae* fam. nov., *Mycoplasmoidales* ord. nov., *Mycoplasmoides* gen. nov., *Mycoplasmopsis* gen. nov. [Gupta, Sawnani, Adeolu, Alnajar and Oren 2018] and all proposed species comb. nov. placed therein', by M. Balish *et al*. (*Int J Syst Evol Microbiol*, 2019;69:3650-3653). Int J Syst Evol Microbiol. (2020) 70:1431–8. doi: 10.1099/ijsem.0.003869, 31971499

[10] Brodersen BW. Bovine respiratory syncytial virus. Vet Clin North Am Food Anim Pract. (2010) 26:323–33. doi: 10.1016/j.cvfa.2010.04.010, 20619187

[11] Jones C Chowdhury S. Bovine herpesvirus type 1 (BHV-1) is an important cofactor in the bovine respiratory disease complex. Vet Clin North Am Food Anim Pract. (2010) 26:303–21. doi: 10.1016/j.cvfa.2010.04.007, 20619186

[12] Brodersen BW. Bovine viral diarrhea virus infections: manifestations of infection and recent advances in understanding pathogenesis and control. Vet Pathol. (2014) 51:453–64. doi: 10.1177/0300985813520250, 24476940

[13] Ellis JA. Bovine parainfluenza-3 virus. Vet Clin North Am Food Anim Pract. (2010) 26:575–93. doi: 10.1016/j.cvfa.2010.08.002, 21056802

[14] Cernicchiaro N Renter DG White BJ Babcock AH Fox JT. Associations between weather conditions during the first 45 days after feedlot arrival and daily respiratory disease risks in autumn-placed feeder cattle in the United States. J Anim Sci. (2012) 90:1328–37. doi: 10.2527/jas.2011-4657, 22147486

[15] Cernicchiaro N White BJ Renter DG Babcock AH Kelly L Slattery R. Associations between the distance traveled from sale barns to commercial feedlots in the United States and overall performance, risk of respiratory disease, and cumulative mortality in feeder cattle during 1997 to 2009. J Anim Sci. (2012) 90:1929–39. doi: 10.2527/jas.2011-4599, 22247119

[16] Griffin D Chengappa MM Kuszak J McVey DS. Bacterial pathogens of the bovine respiratory disease complex. Vet Clin North Am Food Anim Pract. (2010) 26:381–94. doi: 10.1016/j.cvfa.2010.04.004, 20619191

[17] Sasaki Y Hashimoto K Iki Y Anan T Hayashi J Uematsu M. Associations between calf factors of Japanese black calves arriving at a backgrounding operation and bovine respiratory disease. Prev Vet Med. (2020) 182:105100. doi: 10.1016/j.prevetmed.2020.105100, 32755730

[18] Wilson BK Richards CJ Step DL Krehbiel CR. Best management practices for newly weaned calves for improved health and well-being. J Anim Sci. (2017) 95:2170–82. doi: 10.2527/jas.2016.1006, 28727007

[19] Griffin D. Bovine pasteurellosis and other bacterial infections of the respiratory tract. Vet Clin North Am Food Anim Pract. (2010) 26:57–71. doi: 10.1016/j.cvfa.2009.10.010 20117542

[20] Fulton RW. Viruses in bovine respiratory disease in North America: knowledge advances using genomic testing. Vet Clin North Am Food Anim Pract. (2020) 36:321–32. doi: 10.1016/j.cvfa.2020.02.004, 32451028 PMC7244414

[21] Lazurko MM Erickson NEN Campbell JR Gow S Waldner CL. Vaccine use in Canadian cow-calf herds and opportunities for improvement. Front Vet Sci. (2023) 10:1235942. doi: 10.3389/fvets.2023.1235942, 37621868 PMC10445165

[22] Waldner CL Parker S Campbell JR. Vaccine usage in western Canadian cow-calf herds. Can Vet J. (2019) 60:414–22. 30992598 PMC6417607

[23] Erickson NEN Lacoste S Sniatynski M Waldner C Ellis J. Comparison of virus-neutralizing and virus-specific ELISA antibody responses among bovine neonates differentially primed and boosted against bovine coronavirus. Can Vet J. (2024) 65:250–8. 38434170 PMC10880395

[24] Zhang M Hill JE Alexander TW Huang Y. The nasal viromes of cattle on arrival at western Canadian feedlots and their relationship to development of bovine respiratory disease. Transbound Emerg Dis. (2021) 68:2209–18. doi: 10.1111/tbed.13873, 33031627

[25] Richeson JT Falkner TR. Bovine respiratory disease vaccination: what is the effect of timing? Vet Clin North Am Food Anim Pract. (2020) 36:473–85. doi: 10.1016/j.cvfa.2020.03.013, 32451036

[26] O'Connor AM Hu D Totton SC Scott N Winder CB Wang B . A systematic review and network meta-analysis of injectable antibiotic options for the control of bovine respiratory disease in the first 45 days post arrival at the feedlot. Anim Health Res Rev. (2019) 20:163–81. doi: 10.1017/S1466252320000031, 32081117

[27] Hause BM Huntimer L Falkenberg S Henningson J Lechtenberg K Halbur T. An inactivated influenza D virus vaccine partially protects cattle from respiratory disease caused by homologous challenge. Vet Microbiol. (2017) 199:47–53. doi: 10.1016/j.vetmic.2016.12.024, 28110784 PMC7117347

[28] Vetalytix Canada. Compendium of veterinary products: Beef cattle biological chart. Waterloo, ON:Vetalytix Canada (2025). Available online at: https://vetalytixcanada.cvpservice.com/chartindex/ [Accessed June 30, 2025]

[29] Liu R Sheng Z Huang C Wang D Li F. Influenza D virus. Curr Opin Virol. (2020) 44:154–61. doi: 10.1016/j.coviro.2020.08.004, 32932215 PMC7755673

[30] Hawman DW Tipih T Hodge E Stone ET Warner N McCarthy N . Clade 2.3.4.4b but not historical clade 1 HA replicating RNA vaccine protects against bovine H5N1 challenge in mice. Nat Commun. (2025) 16:655. doi: 10.1038/s41467-024-55546-7, 39809744 PMC11732985

[31] Greninger AL Naccache SN Federman S Yu G Mbala P Bres V . Rapid metagenomic identification of viral pathogens in clinical samples by real-time nanopore sequencing analysis. Genome Med. (2015) 7:99. doi: 10.1186/s13073-015-0220-9, 26416663 PMC4587849

[32] Zhang M Hill JE Fernando C Alexander TW Timsit E van der Meer F . Respiratory viruses identified in western Canadian beef cattle by metagenomic sequencing and their association with bovine respiratory disease. Transbound Emerg Dis. (2019) 66:1379–86. doi: 10.1111/tbed.13172, 30873724 PMC7168561

[33] Lager KM Ng TF Bayles DO Alt DP Delwart EL Cheung AK. Diversity of viruses detected by deep sequencing in pigs from a common background. J Vet Diagn Invest. (2012) 24:1177–9. doi: 10.1177/1040638712463212, 23051826

[34] Sadeghi M Popov V Guzman H Phan TG Vasilakis N Tesh R . Genomes of viral isolates derived from different mosquitos species. Virus Res. (2017) 242:49–57. doi: 10.1016/j.virusres.2017.08.012, 28855097 PMC5665172

[35] Quick J Loman NJ Duraffour S Simpson JT Severi E Cowley L . Real-time, portable genome sequencing for Ebola surveillance. Nature. (2016) 530:228–32. doi: 10.1038/nature16996, 26840485 PMC4817224

[36] Kafetzopoulou LE Efthymiadis K Lewandowski K Crook A Carter D Osborne J . Assessment of metagenomic nanopore and Illumina sequencing for recovering whole genome sequences of chikungunya and dengue viruses directly from clinical samples. Euro Surveill. (2018) 23:pii=1800228. doi: 10.2807/1560-7917.ES.2018.23.50.1800228, 30563591 PMC6299504

[37] Roux S Matthijnssens J Dutilh BE. Metagenomics in virology In: Bamford DH Zuckerman M, editors. Encyclopedia of virology (fourth edition). Oxford: Academic Press (2021). 133–40. doi: 10.1016/B978-0-12-809633-8.20957-6

[38] Mitra N Cernicchiaro N Torres S Li F Hause BM. Metagenomic characterization of the virome associated with bovine respiratory disease in feedlot cattle identified novel viruses and suggests an etiologic role for influenza D virus. J Gen Virol. (2016) 97:1771–84. doi: 10.1099/jgv.0.000492, 27154756 PMC5772826

[39] Ng TF Kondov NO Deng X Van Eenennaam A Neibergs HL Delwart E. A metagenomics and case-control study to identify viruses associated with bovine respiratory disease. J Virol. (2015) 89:5340–9. doi: 10.1128/JVI.00064-15, 25740998 PMC4442534

[40] Ambrose RK Blakebrough-Hall C Gravel JL Gonzalez LA Mahony TJ. Characterisation of the upper respiratory tract virome of feedlot cattle and its association with bovine respiratory disease. Viruses. (2023) 15:455. doi: 10.3390/v15020455, 36851669 PMC9961997

[41] MacKenzie M Argyropoulos C. An introduction to nanopore sequencing: past, present, and future considerations. Micromachines. (2023) 14:459. doi: 10.3390/mi14020459, 36838159 PMC9966803

[42] Jager MC Tomlinson JE Lopez-Astacio RA Parrish CR Van de Walle GR. Small but mighty: old and new parvoviruses of veterinary significance. Virol J. (2021) 18:210. doi: 10.1186/s12985-021-01677-y, 34689822 PMC8542416

[43] Zhang M Hill JE Godson DL Ngeleka M Fernando C Huang Y. The pulmonary virome, bacteriological and histopathological findings in bovine respiratory disease from western Canada. Transbound Emerg Dis. (2020) 67:924–34. doi: 10.1111/tbed.13419, 31715071 PMC7168541

[44] Freeman CN Herman EK Abi Younes J Ramsay DE Erikson N Stothard P . Evaluating the potential of third generation metagenomic sequencing for the detection of BRD pathogens and genetic determinants of antimicrobial resistance in chronically ill feedlot cattle. BMC Vet Res. (2022) 18:211. doi: 10.1186/s12917-022-03269-6, 35655189 PMC9161498

[45] Herman EK Lacoste SR Freeman CN Otto SJG McCarthy EL Links MG . Bacterial enrichment prior to third-generation metagenomic sequencing improves detection of BRD pathogens and genetic determinants of antimicrobial resistance in feedlot cattle. Front Microbiol. (2024) 15:1386319. doi: 10.3389/fmicb.2024.1386319, 38779502 PMC11110911

[46] Olfert ED Cross BM McWilliam AA. Guide to the care and use of experimental animals. 2nd ed: Canadian Council on Animal Care, Ottawa, ON (1993).

[47] Canadian Feedlot Antimicrobial Use and Antimicrobial Resistance Surveillance Program (CFAASP). Bovine respiratory disease (BRD) pathogen antimicrobial resistance (AMR) update 2022 (2023). Available online at: https://cahss.ca/CIPARS/Assets/CFAASP/Documents/Respiratory%20pathogen%20AMR%202022%20Final.pdf [Accessed Aug. 14, 2025]

[48] Hannon SJ Brault SA Otto SJG Morley PS McAllister TA Booker CW . Feedlot cattle antimicrobial use surveillance network: a Canadian journey. Front Vet Sci. (2020) 7:596042. doi: 10.3389/fvets.2020.596042, 33330720 PMC7714776

[49] Allander T Tammi MT Eriksson M Bjerkner A Tiveljung-Lindell A Andersson B. Cloning of a human parvovirus by molecular screening of respiratory tract samples. Proc Natl Acad Sci USA. (2005) 102:12891–6. doi: 10.1073/pnas.0504666102, 16118271 PMC1200281

[50] Wick RR Judd LM Holt KE. Deepbinner: demultiplexing barcoded Oxford nanopore reads with deep convolutional neural networks. PLoS Comput Biol. (2018) 14:e1006583. doi: 10.1371/journal.pcbi.1006583, 30458005 PMC6245502

[51] Jurasz H Pawłowski T Perlejewski K. Contamination issue in viral metagenomics: problems, solutions, and clinical perspectives. Front Microbiol. (2021) 12:12. doi: 10.3389/fmicb.2021.745076, 34745046 PMC8564396

[52] Sui HY Weil AA Nuwagira E Qadri F Ryan ET Mezzari MP . Impact of DNA extraction method on variation in human and built environment microbial community and functional profiles assessed by shotgun metagenomics sequencing. Front Microbiol. (2020) 11:953. doi: 10.3389/fmicb.2020.00953, 32528434 PMC7262970

[53] Wick RR Judd LM Gorrie CL Holt KE. Completing bacterial genome assemblies with multiplex MinION sequencing. Microb Genom. (2017) 3:e000132. doi: 10.1099/mgen.0.000132, 29177090 PMC5695209

[54] De Coster W D’Hert S Schultz DT Cruts M Van Broeckhoven C. NanoPack: visualizing and processing long-read sequencing data. Bioinformatics. (2018) 34:2666–9. doi: 10.1093/bioinformatics/bty149, 29547981 PMC6061794

[55] Wood DE Lu J Langmead B. Improved metagenomic analysis with kraken 2. Genome Biol. (2019) 20:257. doi: 10.1186/s13059-019-1891-0, 31779668 PMC6883579

[56] Goldfarb T Kodali VK Pujar S Brover V Robbertse B Farrell CM . NCBI RefSeq: reference sequence standards through 25 years of curation and annotation. Nucleic Acids Res. (2025) 53:D243–57. doi: 10.1093/nar/gkae1038, 39526381 PMC11701664

[57] Hayes BJ Daetwyler HD. 1000 bull genomes project to map simple and complex genetic traits in cattle: applications and outcomes. Annu Rev Anim Biosci. (2019) 7:89–102. doi: 10.1146/annurev-animal-020518-115024, 30508490

[58] Lu J Rincon N Wood DE Breitwieser FP Pockrandt C Langmead B . Metagenome analysis using the kraken software suite. Nat Protoc. (2022) 17:2815–39. doi: 10.1038/s41596-022-00738-y, 36171387 PMC9725748

[59] Bushnell B. BBTools (2020). Available online at: https://sourceforge.net/projects/bbmap/ [Accessed Aug 14, 2025].

[60] Lu J Breitwieser FP Thielen P Salzberg SL. Bracken: estimating species abundance in metagenomics data. PeerJ Comput Sci. (2017) 3:e104. doi: 10.7717/peerj-cs.104, 40271438 PMC12016282

[61] Li H. Seqtk (2018). Available online at: https://github.com/lh3/seqtk [Accessed June 17, 2020].

[62] Feldgarden M Brover V Gonzalez-Escalona N Frye JG Haendiges J Haft DH . AMRFinderPlus and the reference gene Catalog facilitate examination of the genomic links among antimicrobial resistance, stress response, and virulence. Sci Rep. (2021) 11:12728. doi: 10.1038/s41598-021-91456-0, 34135355 PMC8208984

[63] Seemann T. Abricate (2020). Available online at: https://github.com/tseemann/abricate [Accessed May 28, 2020].

[64] McArthur AG Waglechner N Nizam F Yan A Azad MA Baylay AJ . The comprehensive antibiotic resistance database. Antimicrob Agents Chemother. (2013) 57:3348–57. doi: 10.1128/AAC.00419-13, 23650175 PMC3697360

[65] Alcock BP Huynh W Chalil R Smith KW Raphenya AR Wlodarski MA . CARD 2023: expanded curation, support for machine learning, and resistome prediction at the comprehensive antibiotic resistance database. Nucleic Acids Res. (2023) 51:D690–9. doi: 10.1093/nar/gkac920, 36263822 PMC9825576

[66] Vlasova AN Saif LJ. Bovine coronavirus and the associated diseases. Front Vet Sci. (2021) 8:643220. doi: 10.3389/fvets.2021.643220, 33869323 PMC8044316

[67] Donbraye E McLeod L Chai Z Lacoste SR McCarthy EL Hill JE . Clinical diagnostic sensitivity and specificity of metagenomic sequencing and qPCR for detection of viruses associated with bovine respiratory disease estimated using Bayesian latent class models. Front. Vet. Sci. (2026) 13:1704414. doi: 10.3389/fvets.2026.1704414 PMC12976743 41822221

[68] Plummer M. JAGS: A program for analysis of Bayesian graphical models using Gibbs sampling. In. K. Hornik, F. Leisch and A. Zeileis editors. 3rd International Workshop on Distributed Statistical Computing (DSC 2003); Vienna, Austria. Proceedings of the 3rd international workshop on distributed statistical computing (2003).

[69] Denwood MJ. Runjags: an R package providing interface utilities, model templates, parallel computing methods and additional distributions for MCMC models in JAGS. J Stat Softw. (2016) 71:1–25. doi: 10.18637/jss.v071.i09

[70] Wang X Stelzer-Braid S Scotch M Rawlinson WD. Detection of respiratory viruses directly from clinical samples using next-generation sequencing: A literature review of recent advances and potential for routine clinical use. Rev Med Virol. (2022) 32:e2375. doi: 10.1002/rmv.2375, 35775736 PMC9539958

[71] Zhang M Huang Y Godson DL Fernando C Alexander TW Hill JE. Assessment of metagenomic sequencing and qPCR for detection of influenza D virus in bovine respiratory tract samples. Viruses. (2020) 12:814. doi: 10.3390/v12080814, 32731471 PMC7472010

[72] Werid GM Van TD Miller D Hemmatzadeh F Fulton RW Kirkwood R . Bovine parainfluenza-3 virus detection methods and prevalence in cattle: A systematic review and meta-analysis. Animals. (2024) 14:494. doi: 10.3390/ani14030494, 38338137 PMC10854990

[73] Socha W Larska M Rola J Bednarek D. Occurrence of bovine coronavirus and other major respiratory viruses in cattle in Poland. J Vet Res. (2022) 66:479–86. doi: 10.2478/jvetres-2022-0059, 36846034 PMC9945004

[74] Ellis J Marx J Perumbakkam S West K Gow S Lacoste S . Genealogy of an in-vivo passaged isolate of western Canadian bovine respiratory syncytial virus. Can J Vet Res. (2022) 86:218–28. 35794977 PMC9251804

[75] Makoschey B Berge AC. Review on bovine respiratory syncytial virus and bovine parainfluenza - usual suspects in bovine respiratory disease - a narrative review. BMC Vet Res. (2021) 17:261. doi: 10.1186/s12917-021-02935-5, 34332574 PMC8325295

[76] Lee TL Terrell SP Bartle SJ Apley MD Rethorst D Thomson DU . Current feedlot cattle health and well-being program recommendations in the United States and Canada: the 2014 feedlot veterinary consultant survey. Bovine Pract. (2015) 49:124–31. doi: 10.21423/bovine-vol49no2p124-131

[77] Nobrega D Andres-Lasheras S Zaheer R McAllister T Homerosky E Anholt RM . Prevalence, risk factors, and antimicrobial resistance profile of respiratory pathogens isolated from suckling beef calves to reprocessing at the feedlot: A longitudinal study. Front Vet Sci. (2021) 8:764701. doi: 10.3389/fvets.2021.764701, 34805342 PMC8596561

[78] Walz PH Newcomer BW Riddell KP Scruggs DW Cortese VS. Virus detection by PCR following vaccination of naive calves with intranasal or injectable multivalent modified-live viral vaccines. J Vet Diagn Invest. (2017) 29:628–35. doi: 10.1177/1040638717709039, 28545321

[79] Wilhelm BJ Windeyer C Van Donkersgoed J. Beef cow-calf vaccine knowledge translation and transfer (KTT) project: summary report on producer, veterinarian, and working group surveys regarding vaccine usage and recommendations. Can Vet J. (2023) 64:588–94. 37265803 PMC10204882

[80] Wennekamp TR Waldner CL Parker S Windeyer MC Larson K Campbell JR. Biosecurity practices in western Canadian cow-calf herds and their association with animal health. Can Vet J. (2021) 62:712–8. 34219779 PMC8218943

[81] Walz PH Chamorro MF S MF Passler T van der Meer F A RW. Bovine viral diarrhea virus: an updated American College of Veterinary Internal Medicine consensus statement with focus on virus biology, hosts, immunosuppression, and vaccination. J Vet Intern Med. (2020) 34:1690–706. doi: 10.1111/jvim.15816 32633084 PMC7517858

[82] Chamorro MF Palomares RA. Bovine respiratory disease vaccination against viral pathogens: modified-live versus inactivated antigen vaccines, intranasal versus parenteral, what is the evidence? Vet Clin North Am Food Anim Pract. (2020) 36:461–72. doi: 10.1016/j.cvfa.2020.03.006, 32451035 PMC7244452

[83] Lovato L Inman M Henderson G Doster A Jones C. Infection of cattle with a bovine herpesvirus 1 strain that contains a mutation in the latency-related gene leads to increased apoptosis in trigeminal ganglia during the transition from acute infection to latency. J Virol. (2003) 77:4848–57. doi: 10.1128/jvi.77.8.4848-4857.2003, 12663791 PMC152160

[84] Ellis JA. How efficacious are vaccines against bovine respiratory syncytial virus in cattle? Vet Microbiol. (2017) 206:59–68. doi: 10.1016/j.vetmic.2016.11.030, 28024854

[85] Fulton RW Neill JD Saliki JT Landis C Burge LJ Payton ME. Genomic and antigenic characterization of bovine parainfluenza-3 viruses in the United States including modified live virus vaccine (MLV) strains and field strains from cattle. Virus Res. (2017) 235:77–81. doi: 10.1016/j.virusres.2017.04.009, 28416404 PMC7172726

[86] Erickson NEN Berenik A Lardner H Lacoste S Campbell J Gow S . Evaluation of bovine respiratory syncytial virus (BRSV) and bovine herpesvirus (BHV) specific antibody responses between heterologous and homologous prime-boost vaccinated western Canadian beef calves. Can Vet J. (2021) 62:37–44. 33390597 PMC7739395

[87] American Association of Bovine Practitioners (AABP). AABP Vaccination Guidelines. Ashland, OH:American Association of Bovine Practitioners (2021). Available online at: https://www.aabp.org/committees/resources/VaccGuidelines2021.pdf. [Accessed Aug. 14, 2025]

[88] Klem TB Sjurseth SK Sviland S Gjerset B Myrmel M Stokstad M. Bovine respiratory syncytial virus in experimentally exposed and rechallenged calves; viral shedding related to clinical signs and the potential for transmission. BMC Vet Res. (2019) 15:156. doi: 10.1186/s12917-019-1911-z, 31109324 PMC6528318

[89] Grissett GP White BJ Larson RL. Structured literature review of responses of cattle to viral and bacterial pathogens causing bovine respiratory disease complex. J Vet Intern Med. (2015) 29:770–80. doi: 10.1111/jvim.12597, 25929158 PMC4895424

[90] Martinez DA Newcomer B Passler T Chamorro MF. Efficacy of bovine respiratory syncytial virus vaccines to reduce morbidity and mortality in calves within experimental infection models: A systematic review and meta-analysis. Front Vet Sci. (2022) 9:906636. doi: 10.3389/fvets.2022.906636, 35782561 PMC9245045

[91] Martinez DA Chamorro MF Passler T Huber L Walz PH Thoresen M . The titers, duration, and residual clinical protection of passively transferred nasal and serum antibodies are similar among beef calves that nursed colostrum from vaccinated or unvaccinated dams and were challenged experimentally with bovine respiratory syncytial virus at three months of age. Am J Vet Res. (2022) 83:1–9. doi: 10.2460/ajvr.22.07.0118, 36173761

[92] Ellis J Erickson N Gow S West K Lacoste S Godson D. Infection of calves with in-vivo passaged bovine parainfluenza-3 virus, alone or in combination with bovine respiratory syncytial virus and bovine coronavirus. Can J Vet Res. (2020) 84:163–71. 32801450 PMC7301673

[93] Neill JD Ridpath JF Valayudhan BT. Identification and genome characterization of genotype B and genotype C bovine parainfluenza type 3 viruses isolated in the United States. BMC Vet Res. (2015) 11:112. doi: 10.1186/s12917-015-0431-8, 25976921 PMC4438627

[94] Fulton RW Ridpath JF Burge LJ. Bovine coronaviruses from the respiratory tract: antigenic and genetic diversity. Vaccine. (2013) 31:886–92. doi: 10.1016/j.vaccine.2012.12.006, 23246548 PMC7115418

[95] Saif LJ. Bovine respiratory coronavirus. Vet Clin North Am Food Anim Pract. (2010) 26:349–64. doi: 10.1016/j.cvfa.2010.04.005, 20619189 PMC4094360

[96] Ellis J. What is the evidence that bovine coronavirus is a biologically significant respiratory pathogen in cattle? Can Vet J. (2019) 60:147–52. 30705449 PMC6340311

[97] Gaudino M Moreno A Snoeck CJ Zohari S Saegerman C O'Donovan T . Emerging influenza D virus infection in European livestock as determined in serology studies: are we underestimating its spread over the continent? Transbound Emerg Dis. (2021) 68:1125–35. doi: 10.1111/tbed.13812, 32871031

[98] Zhu YM Yu Z Cai H Gao YR Dong XM Li ZL . Isolation, identification, and complete genome sequence of a bovine adenovirus type 3 from cattle in China. Virol J. (2011) 8:557. doi: 10.1186/1743-422X-8-557, 22188676 PMC3265569

[99] Penzes JJ Soderlund-Venermo M Canuti M Eis-Hubinger AM Hughes J Cotmore SF . Reorganizing the family Parvoviridae: a revised taxonomy independent of the canonical approach based on host association. Arch Virol. (2020) 165:2133–46. doi: 10.1007/s00705-020-04632-4, 32533329

[100] Weber MN Cibulski SP Silveira S Siqueira FM Mosena ACS da Silva MS . Evaluation of the serum virome in calves persistently infected with Pestivirus A, presenting or not presenting mucosal disease. Virus Genes. (2018) 54:768–78. doi: 10.1007/s11262-018-1599-3, 30218293

[101] Sadeghi M Kapusinszky B Yugo DM Phan TG Deng X Kanevsky I . Virome of US bovine calf serum. Biologicals. (2017) 46:64–7. doi: 10.1016/j.biologicals.2016.12.009, 28100412 PMC5654489

[102] Wang M Yan Y Wang R Wang L Zhou H Li Y . Simultaneous detection of bovine rotavirus, bovine parvovirus, and bovine viral diarrhea virus using a gold nanoparticle-assisted PCR assay with a dual-priming oligonucleotide system. Front Microbiol. (2019) 10:2884. doi: 10.3389/fmicb.2019.02884, 31921061 PMC6920155

[103] Tokarz R Sameroff S Hesse RA Hause BM Desai A Jain K . Discovery of a novel nidovirus in cattle with respiratory disease. J Gen Virol. (2015) 96:2188–93. doi: 10.1099/vir.0.000166, 25918239 PMC4681066

[104] Hierweger MM Koch MC Seuberlich T. Bovine polyomavirus 2 is a probable cause of non-suppurative encephalitis in cattle. Pathogens. (2020) 9:620. doi: 10.3390/pathogens9080620, 32751201 PMC7459705

[105] Hatama S Nishida T Kadota K Uchida I Kanno T. Bovine papillomavirus type 9 induces epithelial papillomas on the teat skin of heifers. Vet Microbiol. (2009) 136:347–51. doi: 10.1016/j.vetmic.2008.11.003, 19095383

[106] Guo Y McMullen C Timsit E Hallewell J Orsel K van der Meer F . Genetic relatedness and antimicrobial resistance in respiratory bacteria from beef calves sampled from spring processing to 40 days after feedlot entry. Vet Microbiol. (2020) 240:108478. doi: 10.1016/j.vetmic.2019.108478, 31902491

[107] Klima CL Holman DB Ralston BJ Stanford K Zaheer R Alexander TW . Lower respiratory tract microbiome and resistome of bovine respiratory disease mortalities. Microb Ecol. (2019) 78:446–56. doi: 10.1007/s00248-019-01361-3, 30918994

[108] Holman DB Timsit E Booker CW Alexander TW. Injectable antimicrobials in commercial feedlot cattle and their effect on the nasopharyngeal microbiota and antimicrobial resistance. Vet Microbiol. (2018) 214:140–7. doi: 10.1016/j.vetmic.2017.12.015, 29408026

[109] Zaheer R Lakin SM Polo RO Cook SR Larney FJ Morley PS . Comparative diversity of microbiomes and resistomes in beef feedlots, downstream environments and urban sewage influent. BMC Microbiol. (2019) 19:197. doi: 10.1186/s12866-019-1548-x, 31455230 PMC6712873

[110] Wang F Fu Y Lin Z Zhang B Se J Guo X . Neglected drivers of antibiotic resistance: survival of extended-spectrum beta-lactamase-producing pathogenic *Escherichia coli* from livestock waste through dormancy and release of transformable extracellular antibiotic resistance genes under heat treatment. Environ Sci Technol. (2023) 57:9955–64. doi: 10.1021/acs.est.3c02377 37336722

[111] Padalino B Cirone F Zappaterra M Tullio D Ficco G Giustino A . Factors affecting the development of bovine respiratory disease: A cross-sectional study in beef steers shipped from France to Italy. Front Vet Sci. (2021) 8:627894. doi: 10.3389/fvets.2021.627894, 34262960 PMC8273259

[112] Abi Younes JN Campbell JR Otto SJG Gow SP Woolums AR Jelinski M . Variation in pen-level prevalence of BRD bacterial pathogens and antimicrobial resistance following feedlot arrival in beef calves. Antibiotics. (2024) 13:322. doi: 10.3390/antibiotics13040322, 38666998 PMC11047553

[113] Erickson NEN Ngeleka M Lubbers BV Trokhymchuk A. Changes in the rates of field isolation and antimicrobial susceptibility of bacterial pathogens collected from fall placed feedlot steers between arrival at the feedlot and 90 to 120 days on feed. Bovine Pract. (2017) 51:9.

[114] Centeno-Martinez RE Glidden N Mohan S Davidson JL Fernandez-Juricic E Boerman JP . Identification of bovine respiratory disease through the nasal microbiome. Anim Microbiome. (2022) 4:15. doi: 10.1186/s42523-022-00167-y, 35193707 PMC8862248

[115] Oxford Nanaopore Technologies. (2025). PromethION: Flexible, high-output, real-time sequencing for every lab. Oxford Science Park, UK:Oxford Nanopore Technologies. Available online at: https://nanoporetech.com/resource-centre/promethion-brochure [Accessed June 18, 2025].

[116] McDaneld TG Kuehn LA Keele JW. Evaluating the microbiome of two sampling locations in the nasal cavity of cattle with bovine respiratory disease complex (BRDC). J Anim Sci. (2018) 96:1281–7. doi: 10.1093/jas/sky032, 29659872 PMC6140963

[117] Otto SJG Pollock CM Relf-Eckstein J-A McLeod L Waldner CL. Opportunities for laboratory testing to inform antimicrobial use for bovine respiratory disease: application of information quality value stream maps in commercial feedlots. Antibiotics. (2024) 13:903. doi: 10.3390/antibiotics13090903, 39335076 PMC11428555

[118] Zhang D Lou X Yan H Pan J Mao H Tang H . Metagenomic analysis of viral nucleic acid extraction methods in respiratory clinical samples. BMC Genomics. (2018) 19:773. doi: 10.1186/s12864-018-5152-5, 30359242 PMC6202819

[119] Callanan J Stockdale SR Shkoporov A Draper LA Ross RP Hill C. Biases in viral metagenomics-based detection, cataloguing and quantification of bacteriophage genomes in human faeces, a review. Microorganisms. (2021) 9:524. doi: 10.3390/microorganisms9030524, 33806607 PMC8000950

[120] Pevzner P Vingron M Reidys C Sun F Istrail S. Michael waterman's contributions to computational biology and bioinformatics. J Comput Biol. (2022) 29:601–15. doi: 10.1089/cmb.2022.29066.pp, 35727100

[121] Wang Y Zhao Y Bollas A Wang Y Au KF. Nanopore sequencing technology, bioinformatics and applications. Nat Biotechnol. (2021) 39:1348–65. doi: 10.1038/s41587-021-01108-x, 34750572 PMC8988251

[122] Luo J Meng Z Xu X Wang L Zhao K Zhu X . Systematic benchmarking of nanopore Q20+ kit in SARS-CoV-2 whole genome sequencing. Front Microbiol. (2022) 13:973367. doi: 10.3389/fmicb.2022.973367, 36312982 PMC9612837

