# Supplementary material for: Prevalences of respiratory viruses and bacteria in Western Canadian commercial feedlot calves detected using a single metagenomic sequencing protocol vary during the first two weeks of arrival and by age group
Source: Front Vet Sci. 2026 Feb 5;12:1704412. doi: 10.3389/fvets.2025.1704412 (PMC12917898; doi:10.3389/fvets.2025.1704412)
Supplement: Supplementary file 1 [file Data_Sheet_1.pdf]

Supplementary Material – Additional information and sensitivity analyses

Prevalences of Respiratory Viruses and Bacteria in Western Canadian Commercial Feedlot Calves Detected Using a Single Metagenomic Sequencing Protocol Vary During the First Two Weeks of Arrival and by Age Group

Emmanuel Donbraye, Lianne McLeod, Claire N. Carson, Zhijian Chai, Stacey R. Lacoste, Emily K. Herman, E. Luke McCarthy, Janet E. Hill, Nathan E. N. Erickson, Colleen Pollock, Matthew G. Links, Simon J. G. Otto, Sheryl Gow, Paul Stothard, John R. Campbell and Cheryl L. Waldner

Outline:

A. Additional information on respiratory viruses identified in this analysis

B. Impact of not adjusting for water controls during library preparation and sequencing on differences over time and differences between age groups

C. Impact of adjusting using the median of the water controls vs the mean of the water controls on differences over time and differences between age groups

Table S1. Common names and their taxonomic equivalents of 21 viruses detected by metagenomic sequencing in 760 nasal swabs collected from fall-placed calves and yearlings in 19 commercial western Canadian feedlots/pens at the time of arrival processing and at 14 days on feed (DOF)

Virus name	General Name	Taxonomic Name	Taxonomic ID	Genome size (bp)	Family
BCoV	Bovine coronavirus	*Betacoronavirus 1*	*11128*	30,845	*Coronaviridae*
BRBV	Bovine rhinitis B virus	*Bovine rhinitis B virus*	*693066*	7,556	*Picornaviridae*
IDV	Influenza D virus	*Deltainfluenzavirus influenzae*	*2955744*	12,546	*Orthomyxoviridae*
BPIV-3	Bovine parainfluenza virus 3	*Respirovirus bovis*	*3052729*	15,537	*Paramyxoviridae*
BRSV	Bovine respiratory syncytial virus	*Bovine orthopneumovirus*	*11246*	15,140	*Pneumoviridae*
UBPV-6	Bovine bocaparvovirus 2	*Bocaparvovirus ungulate 6*	*1864484*	5,224	*Parvoviridae*
UCPV-1	Bovine parvovirus 2	*Ungulate copiparvovirus 1*	*172296*	5,610	*Parvoviridae*
UCPV-5	Bosavirus	*Ungulate copiparvovirus 5*	*3052146*	5,610	*Parvoviridae*
BoPV	Bovine papilloma virus	*Bovine papillomavirus*	*10571*	7,480	*Papillomaviridae*
BEV	Bovine enterovirus	*Bovine enterovirus*	*12064*	7,414	*Picornaviridae*
UEPV-1	Bovine parvovirus 3	*Ungulate erythroparvovirus 1*	*3052194*	5,276	*Parvoviridae*
UTPV-1	Bovine hokovirus	*Ungulate tetraparvovirus 1*	*3052771*	5,105	*Parvoviridae*
BNV-1	Bovine nidovirus 1	*Bovine nidovirus 1*	*1986112*	20,261	*Tobaniviridae*
BVDV-2	Bovine viral diarrhea virus 2	*Pestivirus tauri*	*54315*	12,513	*Flaviviridae*
BoTV	Bovine torovirus	*Bovine torovirus*	*74501*	28,475	*Tobaniviridae*
BoPyV-2	Bovine polyomavirus 2	*Bovine polyomavirus 2*	*1578134*	5,085	*Polyomaviridae*
UBPV-1	Bovine parvovirus 1	*Ungulate bocaparvovirus 1*	*3052045*	5,224	*Parvoviridae*
BoHV-1	Bovine herpes virus 1	*Bovine alphaherpesvirus 1*	10320	134,896	*Herpesviridae*
BAdV-3	Bovine adenovirus 3	*Bovine mastadenovirus B*	*10510*	34,446	*Adenoviridae*
ICV	Influenza C virus	*Gammainfluenzavirus influenzae*	*11552*	12,900	*Orthomyxoviridae*
BVDV1	Bovine viral diarrhea virus 1	*Pestivirus bovis*	*11099*	12,573	*Flaviviridae*

The following tables show a sensitivity analysis comparable to those displayed in Tables 2-5, but without adjusting for water controls.

These are included as additional supplementary analyses.

Table S2A. Difference in prevalence of ten viruses associated with respiratory disease detected in 520 nasal swabs collected from fall-placed calves (FPC) in western Canadian commercial feedlots at arrival processing and at 14 days on feed (DOF) – no adjustment for water controls.

Samples from fall-placed calves											
	Sample Time 1: Arrival processing (n = 260)				Sample Time 2: 14 DOF (n = 260)				14 DOF samples compared to arrival samples		
Virus	No. pos. samples	Prevalence T1	95% CI	No. reads	No. pos. samples	Prevalence T2	95% CI	No. reads	Odds ratio	95%CI	*P*-value
Viruses with cutoff for positive status based on BLCM											
**BCoV**	105	40%	14-66%	1,191,940	28	11%	4.1-17%	4,457	0.18	0.07-0.46	**<0.001**
**IDV**	59	23%	0.6-39%	17,596	189	73%	53-92%	422,538	9.07	2.63-31.3	**<0.001**
**BRSV**	22	8.5%	0.6-16%	78	81	31%	11-51%	2,472	4.89	1.49-16.1	**0.01**
**BPIV-3**	21	8.1%	0.3-16%	2,186	44	17%	1.6-32%	61,109	2.32	0.84-6.39	**0.10**
**BoHV-1**	0	0%	0-1.4%	0	7	2.7%	0-5.9%	22	9.81	1.46-∞	**0.02**
Viruses for which there are no BLCM informed cutoffs – Sample positive if ≥1 read											
**BRBV**	164	63%	44-82%	51,250	125	48%	31-65%	4,582	0.54	0.30-0.97	0.04
**UCPV-1**	28	11%	3.2-18%	217	52	20%	10-30%	296	2.07	0.85-5.06	**0.11**
**BAdV-3**	3	1.2%	0-2.3%	3	1	0.4%	0-1.1%	2	0.33	0.03-3.43	0.35
**BVDV-2**	3	1.2%	0-3.4%	4	4	1.5%	0-3.8%	5	1.34	0.65-2.74	0.43
**BVDV-1**	0	0%	0-1.4%	0	0	0%	0-1.4%	0	*Not estimable*		0.99

The cut-offs used to determine virus-positive FPC and YRL were based on ≥1 read for all viruses except BPIV-3 (≥5 reads) and BCoV (≥30 reads).

Abbreviations: BCoV: bovine coronavirus; BRBV: bovine rhinitis B virus; IDV: influenza D virus; BPIV3: bovine parainfluenza virus 3; BAdV3: bovine mastadenovirus 3; BRSV: bovine respiratory syncytial virus; UCPV1: ungulate copiparvovirus 1; BVDV2: bovine viral diarrhea1 virus 2; BoHV1: bovine herpes virus 1; BVDV1:bovine viral diarrhea1 virus 1;No.: number; T1: at arrival; T2: 14 day on feed (DOF); CI: confidence interval; BLCM: Bayesian latent class models. **Values in bold were significant in the primary analysis correcting for the mean of water controls (Table 2).**

Table S2B. Difference in prevalence of ten viruses associated with bovine respiratory disease detected in 240 nasal swabs collected from yearlings (YRL) in western Canadian commercial feedlots at the time of arrival processing and at 14 days on feed (DOF) – no adjustment for water controls.

Samples from yearlings											
	**Sample Time 1: Arrival processing (n = 120)**				**Sample Time 2: 14 DOF (n = 120)**				**14 DOF samples compared to arrival samples**		
Virus	No. pos. samples	Prevalence T1	95% CI	No. reads	No. pos. samples	Prevalence T2	95% CI	No. reads	Odds ratio	95%CI	*P*-value
Viruses with cutoff for positive status based on BLCM											
**BRSV**	50	42%	16-67%	188	72	60%	26-94%	622	2.10	1.30-3.39	**0.002**
**BPIV-3**	24	20%	4.8-35%	333	51	43%	16-69%	1,246	2.96	1.99-4.40	**<0.001**
**IDV**	19	16%	0-38%	126	49	41%	6.2-75%	9,475	3.67	0.84-16.0	0.08
**BCoV**	21	18%	0-37%	2,940	21	18%	0-43%	2,895	1.00	0.39-2.53	0.99
**BoHV-1**	0	0.0%	0-3.0%	0	0	0.0%	0-3.0%	0	*Not estimable*		0.99
Viruses for which there are no BLCM informed cutoffs – Sample positive if ≥1 read											
**BRBV**	64	53%	16-91%	4,537	33	28%	0.7-54%	482	0.33	0.07-1.66	0.18
**BVDV-2**	9	7.5%	0.8-14%	14	20	17%	1.3-32%	50	2.50	1.87-3.26	**<0.001**
**UCPV-1**	5	4.2%	0.4-7.9%	6	9	7.5%	1.8-13%	12	1.86	0.42-8.32	0.41
**BVDV-1**	2	1.7%	0-3.7%	2	4	3.3%	0-7.4%	6	2.03	0.43-9.66	0.37
**BAdV-3**	0	0.0%	0-3.0%	0	2	1.7%	0-3.6%	2	2.43	0.19-∞	0.50

The cut-offs used to determine virus-positive FPC and YRL were based on ≥1 read for all viruses except BPIV-3 (≥5 reads) and BCoV (≥30 reads).

Abbreviations: BCoV: bovine coronavirus; BRBV: bovine rhinitis B virus; IDV: influenza D virus; BPIV3: bovine parainfluenza virus 3; BAdV3: bovine mastadenovirus 3; BRSV: bovine respiratory syncytial virus; UCPV1: ungulate copiparvovirus 1; BVDV2: bovine viral diarrhea1 virus 2; BoHV1: bovine herpes virus 1; BVDV1:bovine viral diarrhea1 virus 1;No.: number; T1: at arrival; T2: 14 day on feed (DOF); CI: confidence interval; BLCM: Bayesian latent class models. **Values in bold were significant in the primary analysis correcting for the mean of water controls (Table 3).**

Table S2C. Differences in prevalence of ten viruses associated with bovine respiratory disease detected in samples collected at arrival processing between fall placed calves (FPC) and yearlings (YRL) in western Canadian feedlots – no adjustment for water controls.

Virus	FPC arrival samples: (n=260)		YRL arrival samples: (n=120)		Arrival samples: YRL compared to FPC		
	Prevalence	95% CI	Prevalence	95% CI	Odds Ratio	95%CI	*P*-value
Viruses with cutoff for positive status based on BLCM							
**BCoV**	40%	14-66%	18%	0-37%	0.31	0.06-1.75	0.19
**IDV**	23%	0.6-39%	16%	0-38%	0.64	0.10-4.26	0.65
**BRSV**	8.5%	0.6-16%	42%	16-67%	7.72	1.78-33.5	**0.01**
**BPIV-3**	8%	0.3-16%	20%	4.8-35%	3.63	0.77-17.1	**0.10**
**BoHV-1**	0%	0-1.4%	0%	0-3.0%	*Not estimable*		0.99
Viruses for which there are no BLCM informed cutoffs – Sample positive if ≥1 read							
**BRBV**	63%	44-82%	53%	16-91%	0.67	0.12-3.72	0.65
**UCPV-1**	11%	3.2-18%	4.2%	0.4-7.9%	0.36	0.11-1.21	0.10
**BVDV-2**	1.2%	0-3.4%	7.5%	0.8-14.1%	6.95	0.79-61.2	0.08
**BAdV-3**	1.2%	0-2.3%	0.0%	0-3.0%	0.56	0-5.25	0.64
**BVDV-1**	0%	0-1.4%	1.7%	0-3.7%	5.27	0.41-∞	0.20

The cut-offs used to determine virus-positive FPC and YRL were based on ≥1 read for all viruses except BPIV-3 (≥5 reads) and BCoV (≥30 reads).

Abbreviations: BCoV: bovine coronavirus; BRBV: bovine rhinitis B virus; IDV: influenza D virus; BPIV3: bovine parainfluenza virus 3; BAdV3: bovine mastadenovirus 3; BRSV: bovine respiratory syncytial virus; UCPV1: ungulate copiparvovirus 1; BVDV2: bovine viral diarrheal virus 2; BoHV1: bovine herpes virus 1; BVDV1:bovine viral diarrheal virus 1;No.: number; T1: at arrival; T2: 14 day on feed (DOF); CI: confidence interval; BLCM: Bayesian latent class models. **Values in bold were significant in the primary analysis correcting for the mean of water controls (Table 4).**

Table S2D. Difference in prevalence of ten viruses associated with respiratory disease detected in samples collected at 14 days on feed (DOF) between fall-placed calves (FPC) and yearlings (YRL) in western Canadian feedlots – no adjustment for water controls.

Virus	FPC 14 DOF samples: (n=260)		YRL 14 DOF samples: (n=120)		14 DOF samples: YRL compared to FPC		
	Prevalence	95% CI	Prevalence	95% CI	Odds Ratio	95%CI	*P*-value
Viruses with cutoff for positive status based on BLCM							
**IDV**	73%	53-92%	41%	6.2-75%	0.26	0.05-1.47	0.13
**BRSV**	31%	11-51%	60%	26-94%	3.31	0.62-17.8	0.16
**BPIV-3**	17%	1.6-32%	43%	16-69%	2.85	0.69-11.7	**0.15**
**BCoV**	11%	4.1-17%	18%	0-43%	1.76	0.27-11.5	0.56
**BoHV-1**	2.7%	0-5.9%	0%	0-3.0%	0.22	0-1.49	0.14
Viruses for which there are no BLCM informed cutoffs – Sample positive if ≥1 read							
**BRBV**	48%	31-65%	28%	0.7-54%	0.41	0.09-1.85	0.25
**UCPV-1**	20%	10-30%	7.5%	1.8-13%	0.32	0.11-0.92	0.04
**BVDV-2**	1.5%	0-3.8%	17%	1.3-32%	12.8	1.97-83.3	**0.01**
**BAdV-3**	0.4%	0-1.1%	1.7%	0-3.6%	4.39	0.45-42.7	0.20
**BVDV-1**	0%	0-1.4%	3.3%	0-7.4%	11.7	1.45-∞	0.02

The cut-offs used to determine virus-positive FPC and YRL were based on ≥1 read for all viruses except BPIV-3 (≥5 reads) and BCoV (≥30 reads).

Abbreviations: BCoV: bovine coronavirus; BRBV: bovine rhinitis B virus; IDV: influenza D virus; BPIV3: bovine parainfluenza virus 3; BAdV3: bovine mastadenovirus 3; BRSV: bovine respiratory syncytial virus; UCPV1: ungulate copiparvovirus 1; BVDV2: bovine viral diarrhea1 virus 2; BoHV1: bovine herpes virus 1; BVDV1:bovine viral diarrhea1 virus 1;No.: number; T1: at arrival; T2: 14 day on feed (DOF); CI: confidence interval; BLCM: Bayesian latent class models. **Values in bold were significant in the primary analysis correcting for the mean of water controls (Table 5).**

The following tables show a sensitivity analysis comparable to those displayed in Tables 2-5, after adjusting for the median of the water controls.

These are included as additional supplementary analyses.

Table S3A. Difference in prevalence of ten respiratory viruses associated with respiratory disease detected in 520 nasal swabs collected from fall-placed calves (FPC) in western Canadian commercial feedlots at arrival processing and at 14 days on feed (DOF) – adjusted for the median of the water controls.

**Samples from fall-placed calves**											
	**Sample Time 1: Arrival processing (n = 260)**				**Sample Time 2: 14 DOF (n = 260)**				**14 DOF samples compared to arrival samples**		
Virus	No. pos. samples	Prevalence T1	95% CI	No. reads	No. pos. samples	Prevalence T2	95% CI	No. reads	Odds ratio	95%CI	*P*-value
Viruses with cutoff for positive status based on BLCM											
**BCoV**	83	32%	11-53%	1,151,740	27	10%	4.2-17%	4,366	0.25	0.09-0.69	**0.01**
**IDV**	49	19%	5.7-32%	17,359	132	51%	35-66%	415,826	4.44	1.90-10.4	**0.001**
**BRSV**	22	8.5%	0.6-16%	78	67	26%	9.4-42%	2,411	3.76	1.32-10.7	**0.01**
**BPIV-3**	12	4.6%	0.7-8.5%	2,120	29	11%	3.1-19%	59,378	2.59	1.24-5.43	**0.01**
**BoHV-1**	0	0%	0-1.4%	0	7	2.7%	0-5.9%	22	9.81	1.46-∞	**0.02**
Viruses for which there are no BLCM informed cutoffs – Sample positive if ≥1 read											
**BRBV**	113	43%	32-55%	50,122	106	41%	28-54%	4,414	0.90	0.54-1.48	0.67
**UCPV-1**	21	8.1%	4.9-11%	206	45	17%	10-24%	282	2.38	1.42-4.00	**0.001**
**BVDV-2**	3	1.2%	0-3.4%	4	4	1.5%	0-3.8%	5	1.33	0.65-2.74	0.43
**BAdV-3**	3	1.2%	0-2.3%	3	1	0.4%	0-1.1%	2	0.33	0.03-3.43	0.35
**BVDV-1**	0	0%	0-1.4%	0	0	0%	0-1.4%	0	*Not estimable*		0.99

The cut-offs used to determine virus-positive FPC and YRL were based on ≥1 read for all viruses except BPIV-3 (≥5 reads) and BCoV (≥30 reads).

Abbreviations: BCoV: bovine coronavirus; BRBV: bovine rhinitis B virus; IDV: influenza D virus; BPIV3: bovine parainfluenza virus 3; BAdV3: bovine mastadenovirus 3; BRSV: bovine respiratory syncytial virus; UCPV1: ungulate copiparvovirus 1; BVDV2: bovine viral diarrhea1 virus 2; BoHV1: bovine herpes virus 1; BVDV1:bovine viral diarrhea1 virus 1;No.: number; T1: at arrival; T2: 14 day on feed (DOF); CI: confidence interval; BLCM: Bayesian latent class models. **Values in bold were significant in the primary analysis correcting for the mean of water controls (Table 2).**

Table S3B. Difference in prevalence of ten respiratory viruses associated with bovine respiratory disease detected in 240 nasal swabs collected from yearlings (YRL) in western Canadian commercial feedlots at the time of arrival processing and at 14 days on feed (DOF) – adjusted for the median of the water controls.

**Samples from yearlings**											
	**Sample Time 1: Arrival processing (n = 120)**				**Sample Time 2: 14 DOF (n = 120)**				**14 DOF samples compared to arrival samples**		
Virus	No. pos. samples	Prevalence T1	95% CI	No. reads	No. pos. samples	Prevalence T2	95% CI	No. reads	Odds ratio	95%CI	*P*-value
Viruses with cutoff for positive status based on BLCM											
**BRSV**	47	39%	15-63%	174	72	60%	26-94%	622	2.33	1.44-3.78	**0.001**
**BPIV-3**	24	20%	4.8-35%	333	47	39%	14-65%	1,180	2.56	1.80-3.68	**<0.001**
**IDV**	19	16%	0-38%	126	40	33%	5.3-61%	8,729	2.66	0.47-14.9	0.27
**BCoV**	15	13%	0-23%	2,644	21	18%	0-43%	2,807	1.61	0.57-4.50	0.37
**BoHV-1**	0	0.0%	0-3.0%	0	0	0.0%	0-3.0%	0	*Not estimable*		0.99
Viruses for which there are no BLCM informed cutoffs – Sample positive if ≥1 read											
**BRBV**	50	42%	14-70%	4,338	32	27%	1.3-52%	463	0.51	0.13-2.01	0.34
**BVDV-2**	9	7.5%	0-14%	14	20	17%	1.3-32%	50	2.47	1.87-3.26	**<0.001**
**UCPV-1**	5	4.2%	0.5-7.9%	6	9	7.5%	1.8-13%	12	1.86	0.42-8.32	0.41
**BVDV-1**	2	1.7%	0.4-3.7%	2	4	3.3%	0.8-7.5%	6	2.03	0.43-9.66	0.37
**BAdV-3**	0	0.0%	0-3.0%	0	2	1.7%	0-3.6%	2	2.43	0.19-∞	0.50

The cut-offs used to determine virus-positive FPC and YRL were based on ≥1 read for all viruses except BPIV-3 (≥5 reads) and BCoV (≥30 reads).

Abbreviations: BCoV: bovine coronavirus; BRBV: bovine rhinitis B virus; IDV: influenza D virus; BPIV3: bovine parainfluenza virus 3; BAdV3: bovine mastadenovirus 3; BRSV: bovine respiratory syncytial virus; UCPV1: ungulate copiparvovirus 1; BVDV2: bovine viral diarrhea1 virus 2; BoHV1: bovine herpes virus 1; BVDV1:bovine viral diarrhea1 virus 1;No.: number; T1: at arrival; T2: 14 day on feed (DOF); CI: confidence interval; BLCM: Bayesian latent class models. **Values in bold were significant in the primary analysis correcting for the mean of water controls (Table 3).**

Table S3C. Difference in prevalence of ten respiratory viruses detected in samples collected at arrival processing between yearlings (YRL) and fall placed calves (FPC) in western Canadian commercial feedlots – adjusted for the median of the water controls.

Virus	FPC 14 DOF samples: (n=260)		YRL 14 DOF samples: (n=120)		14 DOF samples: YRL compared to FPC		
	Prevalence	95% CI	Prevalence	95% CI	Odds Ratio	95%CI	*P*-value
Viruses with cutoff for positive status based on BLCM							
**BCoV**	32%	11-53%	13%	0-23%	0.28	0.06-1.27	0.10
**IDV**	19%	5.7-32%	16%	0-38%	0.81	0.13-5.15	0.82
**BRSV**	8.5%	0.6-16%	39%	15-63%	6.97	1.66-29.3	**0.01**
**BPIV-3**	4.6%	0.7-8.5%	20%	4.8-35%	5.17	1.42-18.3	**0.01**
**BoHV-1**	0%	0-1.4%	0%	0-3.0%	*Not estimable*		>0.99
Viruses for which there are no BLCM informed cutoffs – Sample positive if ≥1 read							
**BRBV**	43%	32-55%	42%	14-70%	0.93	0.27-3.20	0.91
**UCPV-1**	8.1%	4.9-11%	4.2%	0.5-7.9%	0.49	0.18-1.37	0.18
**BVDV-2**	1.2%	0-3.4%	7.5%	0-14%	6.95	0.79-61.2	0.08
**BAdV-3**	1.2%	0-2.3%	0.0%	0-3.0%	0.56	0-5.25	0.64
**BVDV-1**	0%	0-1.4%	1.7%	0.4-3.7%	5.27	0.41-∞	0.20

The cut-offs used to determine virus-positive FPC and YRL were based on ≥1 read for all viruses except BPIV-3 (≥5 reads) and BCoV (≥30 reads).

Abbreviations: BCoV: bovine coronavirus; BRBV: bovine rhinitis B virus; IDV: influenza D virus; BPIV3: bovine parainfluenza virus 3; BAdV3: bovine mastadenovirus 3; BRSV: bovine respiratory syncytial virus; UCPV1: ungulate copiparvovirus 1; BVDV2: bovine viral diarrhea1 virus 2; BoHV1: bovine herpes virus 1; BVDV1:bovine viral diarrhea1 virus 1;No.: number; T1: at arrival; T2: 14 day on feed (DOF); CI: confidence interval; BLCM: Bayesian latent class models. **Values in bold were significant in the primary analysis correcting for the mean of water controls (Table 4).**

Table S3D. Difference in prevalence of ten viruses associated with respiratory disease detected in samples collected at 14 days on feed (DOF) between fall-placed calves (FPC) and yearlings (YRL) in western Canadian feedlots – adjusted for the median of the water controls.

Virus	FPC 14 DOF samples: (n=260)		YRL 14 DOF samples: (n=120)		14 DOF samples: YRL compared to FPC		
	Prevalence	95% CI	Prevalence	95% CI	Odds Ratio	95%CI	*P*-value
Viruses with cutoff for positive status based on BLCM							
**IDV**	51%	35-66%	33%	5.3-61%	0.48	0.12-1.98	0.31
**BRSV**	26%	9.4-42%	60%	26-94%	4.32	0.83-22.5	0.08
**BPIV-3**	11%	3.1-19%	39%	14-65%	5.13	1.34-19.7	**0.02**
**BCoV**	10%	4.2-17%	18%	0-43%	1.83	0.28-11.8	0.53
**BoHV-1**	2.7%	0-5.9%	0%	0-3.0%	0.22	0-1.49	0.99
Viruses for which there are no BLCM informed cutoffs – Sample positive if ≥1 read							
**BRBV**	41%	28-54%	27%	1.3-52%	0.53	1.30-2.16	0.38
**UCPV-1**	17%	10-24%	7.5%	1.8-13%	0.39	0.15-1.01	0.051
**BVDV-2**	1.5%	0-3.8%	17%	1.3-32%	12.8	1.97-83.3	**0.01**
**BAdV-3**	0.4%	0-1.1%	1.7%	0-3.6%	4.39	0.45-42.7	0.20
**BVDV-1**	0%	0-1.4%	3.3%	0.8-7.5%	2.03	0.43-9.66	0.37

The cut-offs used to determine virus-positive FPC and YRL were based on ≥1 read for all viruses except BPIV-3 (≥5 reads) and BCoV (≥30 reads).

Abbreviations: BCoV: bovine coronavirus; BRBV: bovine rhinitis B virus; IDV: influenza D virus; BPIV3: bovine parainfluenza virus 3; BAdV3: bovine mastadenovirus 3; BRSV: bovine respiratory syncytial virus; UCPV1: ungulate copiparvovirus 1; BVDV2: bovine viral diarrhea1 virus 2; BoHV1: bovine herpes virus 1; BVDV1:bovine viral diarrhea1 virus 1;No.: number; T1: at arrival; T2: 14 day on feed (DOF); CI: confidence interval; BLCM: Bayesian latent class models. **Values in bold were significant in the primary analysis correcting for the mean of water controls (Table 5).**
